# Physical Training vs. Perindopril Treatment on Arterial Stiffening of Spontaneously Hypertensive Rats: A Proteomic Analysis and Possible Mechanisms

**DOI:** 10.3390/biomedicines11051381

**Published:** 2023-05-06

**Authors:** Danyelle Siqueira Miotto, Francine Duchatsch, Aline Dionizio, Marília Afonso Rabelo Buzalaf, Sandra Lia Amaral

**Affiliations:** 1 Joint Graduate Program in Physiological Sciences (PIPGCF), Federal University of Sao Carlos and São Paulo State University, UFSCar/UNESP, São Carlos 14801-903, Brazil; dany.miotto21@gmail.com (D.S.M.); francine.duchatsch@gmail.com (F.D.); 2 Department of Biological Sciences, Bauru School of Dentistry, University of São Paulo—USP, Bauru 17012-901, Brazil; stars_line@hotmail.com (A.D.);; 3 Department of Physical Education, School of Sciences, São Paulo State University—UNESP, Bauru 17033-360, Brazil

**Keywords:** SHR, pulse wave velocity, perindopril, collagen and aerobic training

## Abstract

(1) Background: Arterial stiffness is an important predictor of cardiovascular events. Perindopril and physical exercise are important in controlling hypertension and arterial stiffness, but the mechanisms are unclear. (2) Methods: Thirty-two spontaneously hypertensive rats (SHR) were evaluated for eight weeks: SHR_C_ (sedentary); SHR_P_ (sedentary treated with perindopril—3 mg/kg) and SHR_T_ (trained). Pulse wave velocity (PWV) analysis was performed, and the aorta was collected for proteomic analysis. (3) Results: Both treatments determined a similar reduction in PWV (−33% for SHR_P_ and −23% for SHR_T_) vs. SHR_C_, as well as in BP. Among the altered proteins, the proteomic analysis identified an upregulation of the EH domain-containing 2 (EHD2) protein in the SHR_P_ group, required for nitric oxide-dependent vessel relaxation. The SHR_T_ group showed downregulation of collagen-1 (COL1). Accordingly, SHR_P_ showed an increase (+69%) in the e-NOS protein level and SHR_T_ showed a lower COL1 protein level (−46%) compared with SHR_C_. (4) Conclusions: Both perindopril and aerobic training reduced arterial stiffness in SHR; however, the results suggest that the mechanisms can be distinct. While treatment with perindopril increased EHD2, a protein involved in vessel relaxation, aerobic training decreased COL1 protein level, an important protein of the extracellular matrix (ECM) that normally enhances vessel rigidity.

## 1. Introduction

An important predictor of cardiovascular events is arterial stiffness, which is assessed by pulse wave velocity (PWV). It is present in hypertension and has been considered an index of vascular aging [1,2,3,4]. In this sense, the imbalance between collagen and elastin, the rupture of elastic fibers, and alterations in several proteins of the vascular components contribute to increased arterial stiffening [5,6,7,8,9].

Both pharmacological and non-pharmacological treatments are promising; however, the mechanisms are still uncertain. Among antihypertensive drugs, it has been shown that perindopril, a renin–angiotensin system (RAS) inhibitor, has higher effectiveness, lower mortality rate among others from the same class, and prevents vascular remodeling [10,11,12], which helps to reduce arterial stiffening when compared to others drugs [13,14]. Recently, our group [15] demonstrated that perindopril reduces blood pressure and does improvements in the RhoA/Rho-kinase/LIMK/Cofilin-1 pathway.

Likewise, physical exercise is a globally accepted tool to control hypertension and PWV [16,17,18], mainly by adjusting the structural components of the vessel wall such as subendothelial matrix proteins and elastic fibers [19].

This study used proteomic analysis to provide a deeper understanding of vascular wall modulations under perindopril treatment and exercise training. Our hypothesis was that both treatments would be effective in controlling arterial stiffening, but probably through different mechanisms.

## 2. Materials and Methods

Thirty-two spontaneously hypertensive rats (SHR, 200–250 g, 2 months old) were obtained from the Animal Facility of the Institute of Biomedical Sciences, University of São Paulo, (USP) Brazil. All rats were housed in the animal facility maintenance at the School of Sciences, São Paulo State University—UNESP, campus of Bauru. All rats received water and food (Biobase, Águas Frias, SC, Brazil) ad libitum and were maintained in a dark–light cycle (12–12 h) and temperature-controlled room (22 ± 2 °C). The animal study protocol was approved by the Committee for Ethical Use of Animals (CEUA) of School of Sciences (UNESP, Bauru, #1320/2019 Vol. 1) and are in accordance with the Brazilian Ethical Principles in Animal Research.

### 2.1. Pharmacological Protocol

All rats were separated into 3 groups with similar body weight (BW) and randomly assigned to undergo an experimental protocol through 8 weeks: SHR_C_ (*n =* 12): sedentary SHR, daily treated with filtered water; SHR_P_ (*n =* 10): sedentary SHR, daily treated with perindopril (Conversyl, 3 mg/kg of BW, via gavage); SHR_T_ (*n =* 10): aerobic-trained SHR throughout the experimental protocol.

The dose of perindopril was chosen based on previous publications [17]. In order to test the effectiveness of the pharmacological treatment, a bolus of Angiotensin I was infused after the treatment period (100 μL, at a dose of 1 μg/μL, i.v.) in 2 treated and 2 control rats and the blood pressure (BP) response was evaluated.

### 2.2. Physical Training

After 7–10 days adaptation period on the treadmill (5–10 min, at 0.3–0.5 km/h), a maximum physical capacity test (Tmax) was performed on all rats, as published [20]. Then, the trained group was subjected to a physical training program on a treadmill (1 h/day, 5 days/week, at 50–60% of their maximum capacity). The Tmax was repeated after 4 weeks for the adjustment of the speed and at the end to evaluate the effects of training [17].

### 2.3. Pulse Wave Velocity

At the end of the experimental protocol, PWV was assessed, as previously published [8,15]. In summary, each rat was anesthetized with xylazine hydrochloride (Anasedan^®^, 10 mg/kg, i.p) and ketamine hydrochloride (Dopalen^®^, 50 mg/kg, i.p), and two pOpet^®^ probes (Axelife SAS, Saint Nicolas de Redon, France) were positioned on the right forelimb (close to elbow) and hindlimb (close to knee). After stabilization of the signal (in a quiet room), the transit time (TT, ms) was recorded for 10 s and registered using pOpet 1.0 software. Taking together the distance between probes (D, cm) and TT, PWV was calculated using the following formula, as previously published [8]:

(1) PWV (m/s) = D (m) /TT (s)

For PWV values, 10 measurements of each rat were obtained, and the average was calculated.

### 2.4. Arterial Pressure

Twenty-four hours after the PWV measurement, all rats were anesthetized with xylazine hydrochloride (Anasedan^®^, 10 mg/mL, i.p) and ketamine hydrochloride (Dopalen^®^, 50 mg/kg, i.p) and the carotid artery was catheterized, as previously published [21]. On the next day, the blood pressure of each awake animal was continuously recorded for at least 1 h, in a quiet room, using a pressure transducer (DPT100, Utah Medical Products Inc., Midvale, UT, USA) connected to the artery cannula, that sent the signal to an amplifier (Quad Bridge Amp, ADInstruments, Colorado Springs, CO, USA) and then to an acquisition board (Powerlab 4/35, ADInstruments, New South Wales, Australia), as published [22]. Systolic blood pressure (SBP) was derived from pulsatile AP recordings using computer software (Labchart pro v7.1, ADInstruments New South Wales, Australia).

### 2.5. Proteomic Analysis

#### 2.5.1. Protein Extraction

After the cardiovascular parameter measurements, all rats were deeply anesthetized by an overload of xylazine hydrochloride and ketamine hydrochloride (Anasedan^®^, 20 mg/kg and Dopalen^®^, 160 mg/kg, i.v., respectively) and euthanized by guillotine. The thoracic aorta was excised, cleaned with saline solution, and homogenized in liquid nitrogen to prevent protein degradation. For the extraction, a total of 50 mg of tissue was homogenized in 500 μL of lysis buffer (7 M urea, 2 M thiourea, and 40 mM Dithiothreitol, all diluted in 50 mM of AMBIC solution) for 2 h in the refrigerator with continuous shaking and, in the end, centrifuged at 20,817× *g* for 30 min at 4 °C, after which the supernatant was taken. Total protein was quantified using the Quick Start ™ Bradford Protein Assay kit (Bio-Rad, Hercules, CA, USA) in duplicate, as described in the literature [23].

#### 2.5.2. Proteomic Analysis of the Aorta

The proteomic analysis was performed as previously described [15,24,25]. A pooled sample of the aorta from 2 rats was analyzed and the proteomic analysis was performed in biological triplicates. They were subdivided into 50 μL aliquots containing 50 μg of proteins (1 μg/μL) and then 25 μL of a 0.2% RapiGest SF solution (Waters Corporation, Milford, MA, USA) was added, followed by agitation and another addition of 10 μL 50 mM of AMBIC. The samples were incubated at 37 °C for 30 min, after which the samples were reduced using 5 mM of dithiothreitol (DTT, Merck KGaA, Darmstadt, Germany), incubated at 37 °C for 40 min and alkylated with 10 mM of iodoacetamide (IAA, Sigma-Aldrich, Darmstadt, Germany), agitated and incubated in the dark at room temperature for 30 min. The samples were digested with the addition of 2% (*w/w*) trypsin (Thermo Scientific, Santa Clara, CA, USA) at 37 °C overnight. After the digestion, 10 μL of 5% trifluoroacetic acid (TFA) was added, followed by agitation and incubation at 37 °C for 90 min. Subsequently, the samples were centrifuged at 20,817× *g* at 6 °C for 30 min. The supernatants were purified and desalinated using a Pierce C18 Spin column (Thermo Scientific, Santa Clara, CA, USA). The supernatant was resuspended in 3% acetonitrile and 0.1% formic acid as standard. The peptide identification was performed on a nanoAcquity UPLC-Xevo QTof MS system (Waters Corporation, Manchester, United Kingdom), as previously described [26]. Protein identification and quantification were obtained using ProteinLynx Global Server (PLGS) version 3.0, using the ion-counting algorithm incorporated into the software. The data obtained were searched in the database of the species Rattus Norvegicus (UniProtKB/Swiss-Prot). The protein profile was obtained using the CYTOSCAPE^®^ software v.3.7.0 (Java^®^ 1.8.0_162) and the plugins ClusterMarker and ClueGO. All proteins identified by the mass spectrometer were inserted into the software, using their access number, and can also be seen in the UniProt database, free of charge available on the virtual platform (Uniprot 2022). After confirming the proteins in the Uniprot_acession database, the first network was created in STRING CONSORTIUM 2022 (STRING version 11.5). Then, it was necessary to make a filter with the taxonomy used in this study (Rattus norvegicus; 10116).

Within this classification, proteins were separated with a ratio value greater than 1 for those found to be upregulated, or with a ratio less than 1 for downregulated. Different numbers were assigned to identify the proteins specific to each group in the comparison.

### 2.6. Protein Analysis of the Aorta

From the aorta, 30 μg of protein was electrophoretically size-separated by using a polyacrylamide gel system (12%) in a running buffer solution for 55 min at 200 V/500 mA/150 W and then transferred to a nitrocellulose membrane at 120 V/500 mA/150 W for 90 min in a buffer solution. The membranes were stained with Ponceau for verification of the protein bands obtained by electrophoresis and washed in a Tris-buffered saline solution with tween-20 (TBS-T). Membranes were incubated within nonfat dry milk for 15 min in TBS-T solution for 10 min. Using the SNAP i.d. 2.0 Protein Detection system (Merck Millipore, Darmstadt, Germany), the membranes were incubated for 10–30 min in their respective primary antibodies (1% albumin bovine serum, BSA): e-Nos (Anti-eNOS/NOS, BD Transduction Laboratories (biosciences), cat #610297, 1:800), cofilin-1 (cofilin-1 (Ab-3), SAB Signalway Antibody cat#21164, 1:500), p-cofilin-1 (cofilin phosphoSer3, cat#ABP54967, 1:500), Collagen-1 (COL1A1, Antibody, Cell Signaling #84336S, 1:800) and Glyceraldehyde-3-phosphate dehydrogenase (GAPDH FL-335, Santa Cruz Biotechnology, INC #sc-25778, 1:800). Membranes were washed with TBS-T and incubated with their respective secondary antibody: anti-mouse (Polyclonal Peroxidase AffiniPure Goat Anti-Mouse IgG, Jackson ImmunoResearch^®^, #115035003, 1:1000) or anti-rabbit (Polyclonal Peroxidase AffiniPure goat anti-rabbit IgG, Jackson ImmunoResearch^®^, #111035003, 1:1000 or 1:800) according to each source of the primary antibody (diluted in 1% BSA). Then, membranes were washed again with TBS-T. The secondary antibodies were detected using a chemical reaction with enhanced luminescence (Immobilon^®^ Crescendo western HRP substrate, Millipore cat#WBLUR0500), and the blots were visualized on C- Digit, Blot Scanner, Li-Cor. The bands were analyzed by using the software Image Studio Digits v. 5.2. The values were normalized by the amount of GAPDH and presented as % of the control group.

### 2.7. Statistical Analysis

All values were presented as mean ± standard error of the mean (SEM). Shapiro–Wilk test was used to test data for normality. For the samples with normal distribution, a one-way analysis of variance (ANOVA) was performed. When the data failed the normality test, we used the transform command in the SigmaStat software to adjust them to meet the normality requirements. All data were analyzed using Sigma Stat software (v4.0.0.37). Tukey’s test was used for the necessary post hoc analysis (*p* < 0.05).

For the proteomic analysis, the comparison between groups was obtained using the Student *t*-test in the PLGS software, considering *p* < 0.05 for the significantly expressed proteins.

## 3. Results

All groups presented similar BW at the beginning of the experimental protocol (252 ± 22, 258 ± 28, and 245 ± 16 g for SHR_C_, SHR_P,_ and SHR_T_, respectively; *p* > 0.05) and similar gain during the eight weeks, since the final BW was similar (307 ± 32, 311 ± 44, and 309 ± 21 g for SHR_C_, SHR_P,_ and SHR_T_, respectively; *p* > 0.05). The maximal physical capacity (seconds during Tmax) was similar between the groups at the beginning (819 ± 244, 789 ± 288, and 726 ± 333 s for SHR_C_, SHR_P,_ and SHR_T_, respectively; *p* > 0.05). The trained rats ran during the first four weeks at a speed of 1.02 km/h (60% of max). After the second Tmax, the treadmill speed was increased by 1.65 km/h in order to maintain the intensity. At the end of eight weeks, the trained group had higher Tmax compared with the control and perindopril groups (577 ± 189, 743 ± 252, and 1511 ± 432 s for SHR_C_, SHR_P,_ and SHR_T_, respectively; *p* < 0.0001). After eight weeks of training or perindopril treatment, the values of SBP (206 ± 10, 131 ± 5, and 150 ± 6 mmHg for SHR_C_, SHR_P,_ and SHR_T_, respectively; *p* < 0.0001), MBP (184 ± 11, 115 ± 4 and 130 ± 7 mmHg for SHR_C_, SHR_P,_ and SHR_T_, respectively; *p* < 0.0001) and DBP (175 ± 12, 108 ± 5 and 120 ± 8 mmHg for SHR_C_, SHR_P,_ and SHR_T_, respectively; *p* = 0.0001) were lower than the control group.

### Arterial Stiffness

**Figure 1:**
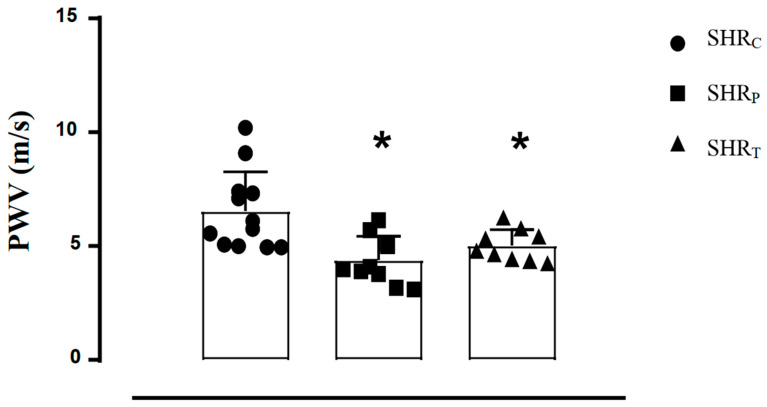
Pulse wave velocity (PWV) values of all SHR groups: sedentary control (SHR_C_, *n =* 12), perindopril-treated sedentary (SHR_P_, *n =* 10), and trained control (SHR_T_, *n =* 10). Significance: * vs. SHR_C_, *p* < 0.05.

As shown in Figure 1, both SHR_P_ (−33%) and SHR_T_ (−23%) groups presented lower PWV values, compared with SHR_C_. There was no difference between the perindopril-treated and trained rats. 

**Figure 2:**
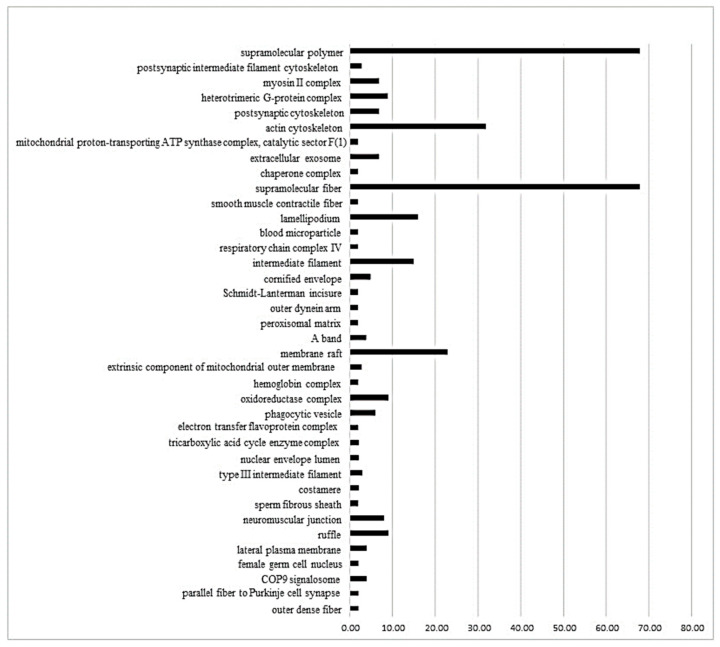
The ClueGo^®^ analysis, comparing SHR_P_ vs. SHR_C_ (Figure 2), demonstrates that 38 subcategories of the cellular component category were modulated. Some categories, such as supramolecular fiber, actin cytoskeleton, supramolecular polymer, and membrane raft, had the highest modulation. The categories are presented and based on the gene ontology according to the cellular component in which they participate, provided by the Cytoscape^®^ software v.3.7.0. Only significant terms were used, and the distribution was made according to the percentage of genes associated with each category. The protein access numbers were made available by UniProt.

The ClueGo^®^ analysis, comparing SHR_P_ vs. SHR_C_ (Figure 2), demonstrates that 38 subcategories of the cellular component category were modulated. Among them, some subcategories, such as supramolecular fiber, actin cytoskeleton, supramolecular polymer, and membrane raft had the highest modulation. As shown in the Supplementary Materials, the process biologic category included 67 modulated subcategories, and the most modulated were the structural constituent of the cytoskeleton, energy derivation by the oxidation of organic compounds, supramolecular fiber organization and response to reactive oxygen species (Table S1, Supplementary Materials). Finally, in the immune system category (Figure S1, Supplementary Materials), only five subcategories were modulated: mature B cell differentiation involved in immune response, T-helper 1 cell differentiation, regulation of T cell-mediated cytotoxicity, negative regulation of myeloid leukocyte mediated immunity and dendritic cell chemotaxis.

**Figure 3:**
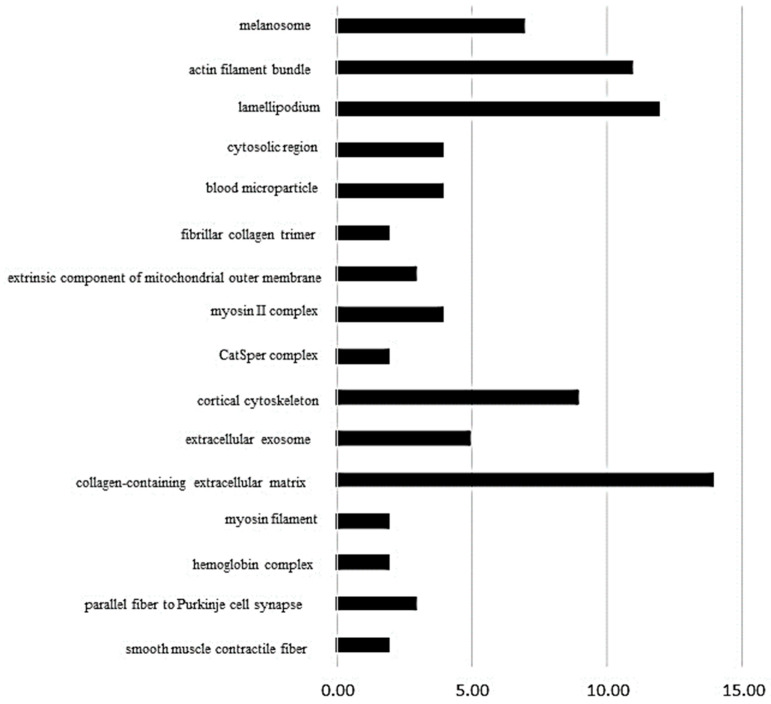
The ClueGo^®^ analysis, comparing SHR_T_ vs. SHR_C_ (Figure 3), demonstrated that 16 subcategories were modulated in the cellular component category and the highest modulated subcategory was collagen-containing extracellular matrix, followed by lamellipodium, actin filament bundle, and cortical cytoskeleton. The categories are presented and based on the gene ontology according to the cellular component in which they participate, provided by the Cytoscape^®^ software v.3.7.0. Only significant terms were used, and the distribution was made according to the percentage of genes associated with each category. The protein access numbers were made available by UniProt.

On the other hand, the ClueGo^®^ analysis, comparing SHR_T_ vs. SHR_C_ (Figure 3), demonstrated that 16 subcategories were modulated in the cellular component category and the highest modulated subcategory was collagen-containing extracellular matrix, followed by lamellipodium, actin filament bundle and cortical cytoskeleton. In the process biologic category (Table S2, Supplementary Materials), there were 48 modulated subcategories, such as actomyosin structure organization, response to heat, regulation of reactive oxygen species metabolic process, and myofibril assembly. In the immune system category, only four subcategories were modulated: positive regulation of leukocyte-mediated cytotoxicity, myeloid dendritic cell differentiation, regulation of T cell-mediated cytotoxicity, and negative regulation of regulatory T cell differentiation (Figure S2, Supplementary Materials).

**Figure 4:**
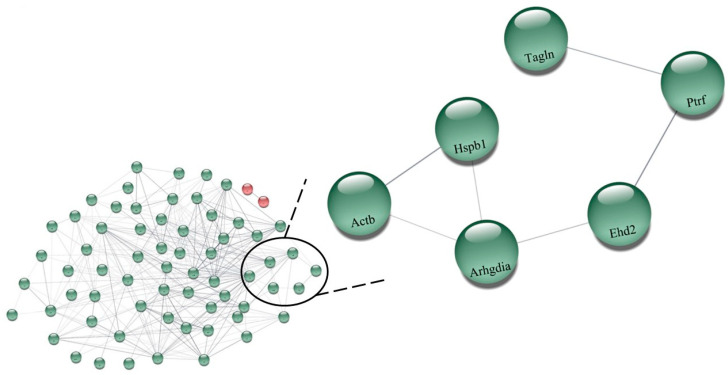
Network illustrating the results between the perindopril-treated rats compared with the control group (SHR_P_ vs. SHR_C_), performed by the CYTOSCAPE^®^ software using the proteins up- and downregulated present in Table S3. The upregulated proteins are in green color: (P36201, Crip2) Cysteine-rich protein 2; (Q6AY56, Tuba8) Tubulin alpha-8 chain; (Q62736, Cald1) Caldesmon 1; (Q4QRB4, Tubb3) Tubulin beta-3 chain; (P31000, Vim) Vimentins; (Q3KRE8, Tubb2b) Tubulin beta-2B chain; (Q00715, Hist1h2bh) Histone cluster 1; (P04636, Mdh2) Malate dehydrogenase, mitochondrial; (P12346, Tf) Serotransferrin; (P47875, Csrp1) Cysteine and glycine-rich protein 1; (P07150, Anxa1) Annexin A1; (Q6P6Q2, Krt5) Keratin, type II cytoskeletal 5;(Q6AYZ1, Tuba1c) Tubulin alpha-1C chain; (P55063, Hspa1l) Heat shock 70 kDa protein 1-like; (P62963, Pfn1) Profilin-1; (P15999, Atp5a1) ATP synthase subunit alpha_ mitochondrial; (P69897, Tubb5) Tubulin beta-5 chain; (Q07936, Anxa2) Annexin A2; (P08010, Gstm2) Glutathione S-transferase Mu 2; (P05065, Aldoa) Fructose-bisphosphate aldolase A; (P63102, Ywhaz) 14-3-3 protein zeta/delta; (Q7M0E3, Dstn) Destrin; (P02454, Col1a1) Collagen alpha-1(I) chain; (Q9Z1P2, Actn1) Alpha-actinin-1; (P63269, Actg2) Actin, gamma-enteric smooth muscle; (Q5XI73, Arhgdia) Rho GDP-dissociation inhibitor 1; (P15650 Acadl) Long-chain specific acyl-CoA dehydrogenase, mitochondrial; (Q9ER34, Aco2) Aconitate hydratase, mitochondrial; (Q5XIF6, Tuba4a) Tubulin alpha-4A chain; (P31232,Tagln) Transgelin; (P18666, Myl12b) Myosin regulatory light chain 12B; (P63018 Hspa8) Heat shock cognate 71 kDa protein; (P85108, Tubb2a) Tubulin beta-2A chain; (P02600, Myl1) Myosin light chain 1/3, skeletal muscle isoform; (Q6P9T8, Tubb4b) Tubulin, beta 4B chain; (P06399, Fga) Fibrinogen alpha chain; (Hspa5) 78 kDa glucose-regulated protein; (P70623, Fabp4) Fatty acid-binding protein 4, adipocyte; (Q68FR8, Tuba3b) Tubulin, alpha 3b; (P21807, Prph) Peripherin; (Q10758, Krt8) Keratin, type II cytoskeletal 8; (Q5RKI0, Wdr1) WD repeat-containing protein 1; (P62630, Eef1a1) Elongation factor 1-alpha 1; (Q9QXQ0, Actn4) Alpha-actinin-4; (P20760, Igg-2a) Ig gamma-2A chain C region; (P68136, Acta1) Actin, alpha skeletal muscle; (Q62812, Myh9) Myosin, heavy chain 9, non-muscle-like 1; (P68035, Acta2) Actin, alpha cardiac muscle 1; (Ptrf) Polymerase 1 and transcript release factor; (P02770, Alb) Serum albumin; (P60711, Actb) Actin, cytoplasmic 1; (Q6IG12, Krt7) Keratin; (P13832, Myl12a) Myosin regulatory light chain RLC-A; (P48675, Des) Desmin; (Q4V8H8, Ehd2) EH domain-containing protein 2; (P47853, Bgn) Biglycan; (Q9WVH8, Fbln5) Fibulin-5; (P14659, Hspa2) Heat shock-related 70 kDa protein 2; (P11762, Lgals1) Galectin-1; (Q9JLT0, Myh10) Myosin heavy chain 9/10/11/14; (P42930, Hspb1) Heat shock protein family b (small) member 1; (P85973, Pnp) Purine-nucleoside phosphorylase; (P68370, Tuba1a) Tubulin alpha-1A chain; (Q64122, Myl9) Myosin regulatory light chain 9; (P10111, Ppia) Peptidyl-prolyl cis-trans isomerase A; (P42930, Hspa1a) Heat shock 70kd protein 1b (mapped); (Q01129, Dcn) Decorin; (P16409, Myl3) Myosin light chain 3. On the other hand, only two proteins were downregulated which are in red: (P01946, Hba1) Hemoglobin subunit alpha-1/2 and (P02091, Hbb) Hemoglobin subunit beta-1 after perindopril treatment. Highlighted: (P60711, Actb) Actin, cytoplasmic 1; (P42930, Hspb1) Heat shock protein family b (small) member 1; (Q5XI73, Arhgdia) Rho GDP-dissociation inhibitor 1; (Q4V8H8, EHD2) EH domain-containing protein 2; (Ptrf) Polymerase 1 and transcript release factor; (P31232, Tagln) Transgelin.

Table S3 (Supplementary Materials) shows all the 138 expressed proteins under the effects of perindopril on hypertension (SHR_P_ vs. SHR_C_). Among them, 73 were upregulated and only 2 were downregulated. Figure 4 illustrates the network performed by the CYTOSCAPE^®^ software using the proteins up- and downregulated present in Table S3 (Supplementary Materials) showing the results between the perindopril-treated rats compared with the control group (SHR_P_ vs. SHR_C_). The upregulated proteins are in green color: (P36201, Crip2) Cysteine-rich protein 2; (Q6AY56, Tuba8) Tubulin alpha-8 chain; (Q62736, Cald1) Caldesmon 1; (Q4QRB4, Tubb3) Tubulin beta-3 chain; (P31000, Vim) Vimentins; (Q3KRE8, Tubb2b) Tubulin beta-2B chain; (Q00715, Hist1h2bh) Histone cluster 1; (P04636, Mdh2) Malate dehydrogenase, mitochondrial; (P12346, Tf) Serotransferrin; (P47875, Csrp1) Cysteine and glycine-rich protein 1; (P07150, Anxa1) Annexin A1; (Q6P6Q2, Krt5) Keratin, type II cytoskeletal 5;(Q6AYZ1, Tuba1c) Tubulin alpha-1C chain; (P55063, Hspa1l) Heat shock 70 kDa protein 1-like; (P62963, Pfn1) Profilin-1; (P15999, Atp5a1) ATP synthase subunit alpha_ mitochondrial; (P69897, Tubb5) Tubulin beta-5 chain; (Q07936, Anxa2) Annexin A2; (P08010, Gstm2) Glutathione S-transferase Mu 2; (P05065, Aldoa) Fructose–bisphosphate aldolase A; (P63102, Ywhaz) 14-3-3 protein zeta/delta; (Q7M0E3, Dstn) Destrin; (P02454, Col1a1) Collagen alpha-1(I) chain; (Q9Z1P2, Actn1) Alpha-actinin-1; (P63269, Actg2) Actin, gamma-enteric smooth muscle; (Q5XI73, Arhgdia) Rho GDP-dissociation inhibitor 1; (P15650 Acadl) Long-chain specific acyl-CoA dehydrogenase, mitochondrial; (Q9ER34, Aco2) Aconitate hydratase, mitochondrial; (Q5XIF6, Tuba4a) Tubulin alpha-4A chain; (P31232,Tagln) Transgelin; (P18666, Myl12b) Myosin regulatory light chain 12B; (P63018 Hspa8) Heat shock cognate 71 kDa protein; (P85108, Tubb2a) Tubulin beta-2A chain; (P02600, Myl1) Myosin light chain 1/3, skeletal muscle isoform; (Q6P9T8, Tubb4b) Tubulin, beta 4B chain; (P06399, Fga) Fibrinogen alpha chain; (Hspa5) 78 kDa glucose-regulated protein; (P70623, Fabp4) Fatty acid-binding protein 4, adipocyte; (Q68FR8, Tuba3b) Tubulin, alpha 3b; (P21807, Prph) Peripherin; (Q10758, Krt8) Keratin, type II cytoskeletal 8; (Q5RKI0, Wdr1) WD repeat-containing protein 1; (P62630, Eef1a1) Elongation factor 1-alpha 1; (Q9QXQ0, Actn4) Alpha-actinin-4; (P20760, Igg-2a) Ig gamma-2A chain C region; (P68136, Acta1) Actin, alpha skeletal muscle; (Q62812, Myh9) Myosin, heavy chain 9, non-muscle-like 1; (P68035, Acta2) Actin, alpha cardiac muscle 1; (Ptrf) Polymerase 1 and transcript release factor; (P02770, Alb) Serum albumin; (P60711, Actb) Actin, cytoplasmic 1; (Q6IG12, Krt7) Keratin; (P13832, Myl12a) Myosin regulatory light chain RLC-A; (P48675, Des) Desmin; (Q4V8H8, Ehd2) EH domain-containing protein 2; (P47853, Bgn) Biglycan; (Q9WVH8, Fbln5) Fibulin-5; (P14659, Hspa2) Heat shock-related 70 kDa protein 2; (P11762, Lgals1) Galectin-1; (Q9JLT0, Myh10) Myosin heavy chain 9/10/11/14; (P42930, Hspb1) Heat shock protein family b (small) member 1; (P85973, Pnp) Purine-nucleoside phosphorylase; (P68370, Tuba1a) Tubulin alpha-1A chain; (Q64122, Myl9) Myosin regulatory light chain 9; (P10111, Ppia) Peptidyl-prolyl cis-trans isomerase A; (P42930, Hspa1a) Heat shock 70kd protein 1b (mapped); (Q01129, Dcn) Decorin; (P16409, Myl3) Myosin light chain 3. On the other side, only two proteins were downregulated which are in red: (P01946, Hba1) Hemoglobin subunit alpha-1/2 and (P02091, Hbb) Hemoglobin subunit beta-1 after perindopril treatment (Figure 4).

**Figure 5:**
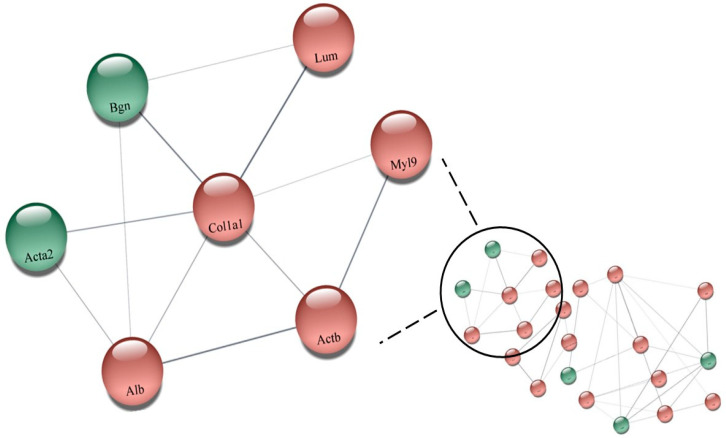
Network illustrating the effects of training on hypertension showed that 123 proteins were differently expressed (Table S4). While 7 of them were upregulated, 22 were downregulated (SHR_T_ vs. SHR_C_), performed by the CYTOSCAPE^®^ software using the proteins up- and downregulated. The upregulated proteins are in green color: (P68035, Acta2) Actin_ alpha cardiac muscle 1; (P63269, Actg2) Actin_ gamma-enteric smooth muscle; (P47853, Bgn) Biglycan; (P06761, Hspa5) Endoplasmic reticulum chaperone BiP; (P70490, Mfge8) Lactadherin; (Q6AY56, Tuba8) Tubulin alpha-8 chain; (Q9JLT0, Myh10) Myosin-10. The downregulated proteins are shown in red: (P47875, Csrp1) Cysteine and glycine-rich protein 1; (Q7M0E3, Dstn) Destrin; (P02454, Col1a1) Collagen alpha-1(I) chain; (P06866, Hp) Haptoglobin; (P50399, Gdi2) Rab GDP dissociation inhibitor beta; (P68136, Acta1) Actin_ alpha skeletal muscle; (P62738) Actin_ aortic smooth muscle; (P60711, Actb) Actin_ cytoplasmic 1; (P63259) Actin_ cytoplasmic 2; (Q9Z1P2, Actn1) Alpha-actinin-1; (P36201, Crip2) Cysteine-rich protein 2; (P04797, Gapdh) Glyceraldehyde-3-phosphate dehydrogenase; (P42930, Hspb1) Heat shock protein beta-1; (P01946, Hba1) Hemoglobin subunit alpha-1/2; (P02091, Hbb) Hemoglobin subunit beta-1; (P11517, ENSRNOP00000048546) Hemoglobin subunit beta-2; (P20760, Igg-2a) Ig gamma-2A chain C region; (P51886, Lum) Lumican; (Q64119, Myl6) Myosin light polypeptide 6; (Q64122, Myl9) Myosin regulatory light polypeptide 9; (P10111, Ppia) Peptidyl-prolyl cis-trans isomerase A; (P02770, Alb) Serum albumin. Highlighted: (Q64122, Myl9) Myosin regulatory light polypeptide 9; (P02454, Col1a1) Collagen alpha-1(I) chain; (P60711, Actb) Actin_ cytoplasmic 1; (P51886, Lum) Lumican; (P02770, Alb) Serum albumin; (P68035, Acta2) Actin_ alpha cardiac muscle 1; (P47853, Bgn) Biglycan.

The comparison between SHR_T_ and SHR_C_, regarding the effects of training on hypertension as shown in Table S4 (Supplementary Materials), showed that 123 proteins were differently expressed. While 7 of them were upregulated, 22 were downregulated. The network made with the proteins in Table S4 (Supplementary Materials) is illustrated in Figure 5 (SHR_T_ vs. SHR_C_). As shown, the upregulated proteins are in green color: (P68035, Acta2) Actin_ alpha cardiac muscle 1; (P63269, Actg2) Actin_ gamma-enteric smooth muscle; (P47853, Bgn) Biglycan; (P06761, Hspa5) Endoplasmic reticulum chaperone BiP; (P70490, Mfge8) Lactadherin; (Q6AY56, Tuba8) Tubulin alpha-8 chain; (Q9JLT0, Myh10) Myosin-10. The downregulated proteins are shown in red: (P47875, Csrp1) Cysteine and glycine-rich protein 1; (Q7M0E3, Dstn) Destrin; (P02454, Col1a1) Collagen alpha-1(I) chain; (P06866, Hp) Haptoglobin; (P50399, Gdi2) Rab GDP dissociation inhibitor beta; (P68136, Acta1) Actin_ alpha skeletal muscle; (P62738) Actin_ aortic smooth muscle; (P60711, Actb) Actin_ cytoplasmic 1; (P63259) Actin_ cytoplasmic 2; (Q9Z1P2, Actn1) Alpha-actinin-1; (P36201, Crip2) Cysteine-rich protein 2; (P04797, Gapdh) Glyceraldehyde-3-phosphate dehydrogenase; (P42930, Hspb1) Heat shock protein beta-1; (P01946, Hba1) Hemoglobin subunit alpha-1/2; (P02091, Hbb) Hemoglobin subunit beta-1; (P11517, ENSRNOP00000048546) Hemoglobin subunit beta-2; (P20760, Igg-2a) Ig gamma-2A chain C region; (P51886, Lum) Lumican; (Q64119, Myl6) Myosin light polypeptide 6; (Q64122, Myl9) Myosin regulatory light polypeptide 9; (P10111, Ppia) Peptidyl-prolyl cis-trans isomerase A; (P02770, Alb) Serum albumin (Figure 5).

**Figure 6:**
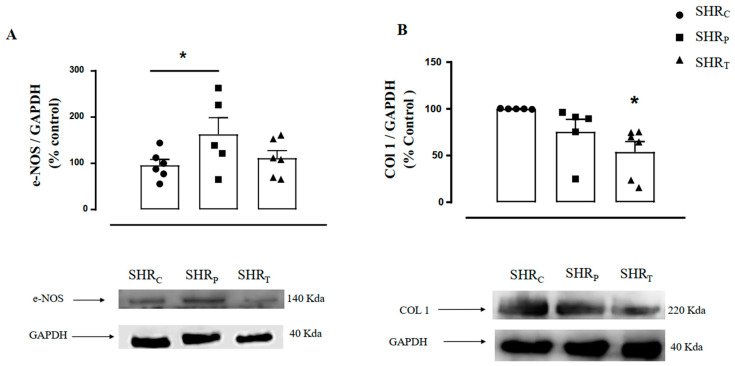
Illustration of the protein level in aortic endothelial nitric oxide synthase protein (e-NOS, (**A**)) and collagen 1 (COL1, (**B**)) protein in all groups: sedentary control SHR (SHR_C_, *n =* 6), sedentary treated with perindopril (SHR_P_, *n =* 6) and trained control (SHR_T_, *n =* 6). Figure 6 (bottom panel) also illustrates the representative Gel Blot of e-NOS and COL1 levels in the aorta of all groups, namely, SHR_C_, SHR_P,_ and SHR_T_, respectively. Significance: * vs. SHR_C_, *p* < 0.05.

Figure 6 illustrates the densitometric analysis of the e-NOS (Figure 6A) and COL1 (Figure 6B) protein levels in the aorta of all rats. As shown, the SHR_P_ group had higher values of aortic e-NOS protein level (+69%) when compared with the control group. Thus, it can be said that only perindopril treatment in SHR was able to increase e-NOS expression, while training did not significantly increase it when compared to the control group.

On the other hand, aortic COL1 level was lower in the SHR_T_ group, compared with the control group (−46%, Figure 6B), suggesting that training was able to reduce COL1 expression in SHR, but treatment with perindopril did not significantly reduce it.

**Figure 7:**
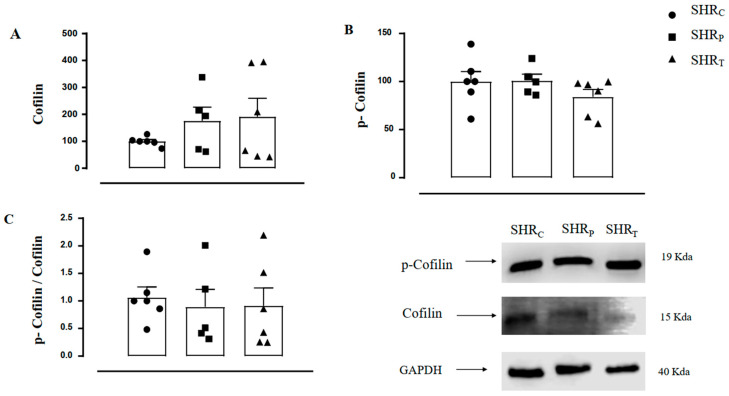
Values of aortic cofilin-1 protein (**A**), p-cofilin-1 protein (**B**), and the p-cofilin-1/cofilin-1 ratio (**C**) in all groups: sedentary control (SHR_C_, *n =* 6), sedentary treated with perindopril (SHR_P_, *n =* 6) and trained control (SHR_T_, *n =* 6). Figure 7 also illustrates the representative Gel Blot of Cofilin-1, p-Cofilin-1, and GAPDH of the aorta in all groups, namely, SHR_C_, SHR_P,_ and SHR_T_, respectively.

The values of aortic cofilin-1 (Figure 7A), p-cofilin-1 (Figure 7B), and the ratio p-cofilin/cofilin-1 (Figure 7C) were similar between the groups, as shown in Figure 7. Therefore, neither perindopril treatment nor aerobic physical training was able to significantly modulate the total and/or phosphorylated cofilin-1 behavior in these SHR animals.

## 4. Discussion

The main results of the present study were that either perindopril or physical training significantly reduced the pulse wave velocity of hypertensive rats. On the other hand, the proteomic analysis indicated that pharmacological and non-pharmacological treatments regulated distinct proteins in the aorta, suggesting that the mechanisms may be different.

Since arterial stiffness has been considered a marker of vessel aging and a predictor for cardiovascular diseases and future events [2,27], there is a strong recommendation to include this measure in clinical practice [28], sometimes even for pediatric routine [29]. Although it is not clear if arterial stiffness precedes hypertension or vice versa [30,31], several studies clearly demonstrate that hypertensive individuals have higher PWV [8,15,32,33]. Therefore, the maintenance of normal values for blood pressure and PWV are the goals suggested by worldwide guidelines for the management of hypertension [34,35,36]. It has been shown that an increase of 1 m/s in PWV induces an increase of 15% in cardiovascular risk [37].

It is well-known that increased activity of the renin–angiotensin system (RAS) increases BP and causes vessel remodeling [13]. Therefore, angiotensin-converting enzyme (ACE) inhibitors are highly recommended, mainly because they alter the structure of vessels beyond BP lowering. Ong et al. [38] compared different antihypertensive drug classes, such as diuretic, beta-blocker, calcium antagonist, and ACEi, on arterial stiffness and BP improvement and concluded that the reduction in arterial stiffness is higher under ACEi than under calcium antagonist in a four-week treatment, while all classes had similar responses after four weeks of treatment. In addition, ACEi allows the circulating bradykinin bioavailability, which contributes to the formation of nitric oxide [39] and induces vasodilation.

Recently, our group has shown that SHR rats had higher BP and PWV, compared with normotensive rats, and eight weeks of perindopril treatment reduced both BP and PWV [17]. Likewise, the results of this present study (Figure 1) confirmed our previous results and showed that perindopril-treated SHR had lower BP and PWV compared with the control SHR.

Physical training has been considered an important adjunct to pharmacological treatment to control hypertension and is highly recommended by hypertension societies around the world [34,35,36], and the mechanism involves a better control of cardiac output and peripheral vascular resistance [40,41,42,43]. In addition, exercise training significantly decreases PWV, and the clinical relevance of different types of exercise on PWV reduction has been shown in several pathologies and hypertension [44,45,46,47,48]. In agreement, this present study showed that eight weeks of aerobic exercise training also reduced BP and PWV, and, interesting to note, both groups, the perindopril, and the trained SHR, presented similar values of BP and PWV compared with the control SHR.

From human studies, most of the mechanisms shown to be involved with PWV reduction are systemic, such as increases in plasma nitrite concentration and plasma NOx [48,49,50] and decreases in plasma levels of endothelin-1 and noradrenaline [48,49]. On the other hand, animal studies have shown important alterations both in the aortic extracellular matrix proteins and in the hypertrophy of vascular smooth muscle cells (VSMC), which contribute to altering vessel remodeling, but not all animal studies evaluate PWV [51]. Therefore, the present study used a non-invasive technique, previously validated by our group [8], which allows measuring PWV and performing histological and molecular analyses in the vessel of the same animal for a better understanding of the possible mechanisms.

The present study carried out a proteomic analysis in the aorta, which allowed the identification of differently expressed proteins of both groups, trained and perindopril-treated SHR. Previously, our group has identified an upregulation of GDP dissociation inhibitor protein (GDIs) in the aorta of perindopril-treated SHR, which is an internal regulator of RhoA pathway activation, suggesting that treated SHR had an inhibition of the RhoA/ROCK/LIMK/Cofilin-1 pathway [15]. Accordingly, Morales-Quinones et al. [52] showed that LIMK inhibition reduces p-Cofilin/Cofilin, which was followed by a reduction in arterial stiffness. Although the results of this present study also showed an upregulation of GDIs after treatment with perindopril followed by a reduction in arterial stiffness, the results of aortic p-Cofilin/Cofilin protein level were only slightly reduced. Probably the higher variability between rats interfered with the results, and this is a limitation of this study.

Additionally, the proteomic analysis showed an interaction between GDIs and EHD2 protein, which was also upregulated in the SHR_P_ group. Cellular homeostasis is maintained due to an organized process of internalization of nutrients and molecules that move along a series of tubular membranes, and this process is known as the endocytic trafficking system [53]. Likewise, this system is necessary for the product’s degradation to return to the membrane surface. Several proteins are recruited to orchestrate this endocytic transport, and among these are the C terminal Eps15 homology domain (EHD)-containing proteins. EHD2 is one of these proteins (family of 4 EHD) that is highly expressed in many tissues including fat, skeletal muscle, lung, spleen, kidney, heart, and tissues rich in caveolae like blood vessels [53,54,55]. It has been shown that this protein is important for the eNOS-NO-dependent vessel relaxation since EHD2 knockout mice present lower NO abundance in the vascular endothelium and impaired acetylcholine-induced relation in mesenteric arteries [54]. Moreover, the proteomic analysis also demonstrated an interaction of the EHD2 protein with the caveolae-associated protein 1 (Ptrf), which is an important protein involved in the formation of caveolae. Ptrf is essential for caveolae recruitment in the presence of caveolin-1. Matthaeus et al. [54] demonstrated that EHD2 knockout mice showed a decrease in NO production, regardless of eNOS levels, which did not change. Furthermore, they showed that, in these mice lacking EHD2, the caveola was detached from the membrane, which resulted in the redistribution of eNOS into the cytoplasm. Indeed, EHD2 knockdown HUVECs showed that detached caveolae still contained eNOS; however, they observed reduced phosphorylation of eNOS Ser1177 in EHD2 knockdown endothelial cells, which was indicative of reduced eNOS activity. Therefore, these authors concluded that EHD2 in the caveolae neck is required for correct eNOS localization and signaling, and therefore for proper endothelial function. Based on this proposition, and on the results of this present study, we hypothesized that the up-regulated EHD2 observed in the proteomic results could be contributing to maintaining the stabilization of the caveolae at the plasma membrane and, in turn, to the correct location and function of eNOS, which could be modulating vessel relaxation in the perindopril group, as demonstrated by reduced PWV. Although the level of aortic eNOS protein was increased in the present study, we did not evaluate its activity. We also did not evaluate NO formation. However, we have previously shown [15] that perindopril treatment increased plasma nitrite concentration (indicative of NO formation) by 83% in SHR, and this response was negatively correlated with PWV. Therefore, we believe that the correct stabilization of caveolae in the plasma membrane, modulated by the level of the EHD2 protein, could orchestrate the localization and activity of eNOS. We may assume that the lower PWV observed in the group treated with perindopril was induced by the eNOS/NO pathway, which was allowed by a correct stabilization of the caveolae in the membrane, induced by the upregulation of EHD2.

Unlike the effects of perindopril, proteomic analysis revealed that aerobic training downregulated the COL1a1 protein in the aorta of SHR. The elastic characteristic of arteries depends on the balance between structural proteins responsible for determining contraction and relaxation, such as collagen and elastin. Any change in these components, such as increased collagen synthesis and deposition, elastin degradation, as well as disruption of elastic fibers, can lead to vessel remodeling and increased stiffness [7,8,32,56]. Collagen (COL1 and COL3) along with elastin are the major extracellular matrix structural proteins of the cardiovascular system. It is widely distributed extracellularly in most tissues. Both collagen types are predominantly secreted by fibroblasts and smooth muscle cells. While COL1 is the main collagen type present in bone, tendons, dermis, ligaments, and connective tissue, COL3 is distributed mostly in the skin, vessel walls and reticular fibers of most tissues [57]. Collagen and elastin have a key role in modulating the tight balance of elasticity, resilience, and rigidity, which is necessary for physiological functions. Since the elastic fiber network is the most distensible component of the arterial wall, and the collagen fiber network provides rigidity and strength of the arterial wall, the vascular balance of COL and elastin is necessary for vessel physiological function [56,58]. In addition, both fibrillar collagens have similar physiological functions, but COL1 is stiffer and provides structural rigidity over COL 3, which is thinner. In this sense, recently, Witting and Szulcek [58] have proposed that the normal physiologic range of the aortic COL1/COL3 ratio is around 2.04 to 3.83, which is in agreement with a recent study from our group [59] and that COL1 increases to collagen-III in all non-physiologic cases, including hypertension and atherosclerosis.

To confirm the proteomic finding, we evaluated the COL1 protein level on the aorta of the trained SHR and observed that the trained group had a 46% lower level of COL1 when compared with the control SHR group. In addition, less aortic collagen level has been demonstrated after aerobic training [19,60] which contributes to decreased arterial stiffening. The mechanism induced by aerobic training to reduce the level of aortic COL1 protein may involve a lower sympathetic drive to the vessel [17,51] since the synthesis of collagen is mediated by increased sympathetic nerve activity through the beta receptor [61].

Additionally, the network performed in this present work indicated that the downregulated COL1a1 protein directly interacted with the protein Lumican, which was also downregulated. Lumican is a proteoglycan of the extracellular matrix involved in collagen fibrillogenesis and changes in its content may affect collagen organization and, consequently, blood vessels’ elastic properties [62]. In this sense, higher expressions of lumican have been found in the aorta of patients with chronic renal failure [63] and in patients with aortic dissection [64]. The reduced regulation of COL1 and Lumican in the aorta of the trained SHR could contribute to decreased vessel rigidity observed in hypertension.

## 5. Conclusions

In conclusion, the present study indicated that both perindopril and aerobic training similarly reduced arterial stiffness in SHR; however, the proteomic analysis on the aorta revealed that the mechanisms can be distinct. While treatment with perindopril increased the EHD2, a protein involved in the vessel relaxation induced by the e-NOS-NO pathway, aerobic training decreased the aortic COL1 protein level, an important protein of the ECM that normally enhances vessel rigidity.

## Data Availability

The data presented in this study are available on request from the corresponding author.

## Supplementary Materials

The following supporting information can be downloaded at: https://www.mdpi.com/article/10.3390/biomedicines11051381/s1, Figure S1: Protein analysis in ClueGO plugins and the number of genes involved within the system immune category between SHR_P_ vs. SHR_C_ groups; Figure S2: Protein analysis in ClueGO plug-ins and the number of genes involved within the sistem immune category between SHR_T_ vs. SHR_C_ groups; Table S1: Protein analysis in ClueGO plugins and the number of genes involved within the process biologic category between SHR_P_ vs. SHR_C_ groups; Table S2: Protein analysis in ClueGO plug-ins and the number of genes involved within the process biological category between SHR_T_ vs SHR_C_ groups; Table S3: The protein of comparison SHR_P_ vs. SHR_C_; Table S4: The protein of comparison SHR_T_ vs. SHR_C_.

## References

[1] Wadstrom B.N., Fatehali A.H., Engstrom G., Nilsson P.M. (2019). A Vascular Aging Index as Independent Predictor of Cardiovascular Events and Total Mortality in an Elderly Urban Population. Angiology.

[2] Rovella V., Gabriele M., Sali E., Barnett O., Scuteri A., Di Daniele N. (2020). Is Arterial Stiffness a Determinant of Hypotension?. High Blood Press. Cardiovasc. Prev..

[3] Valencia-Hernandez C.A., Lindbohm J.V., Shipley M.J., Wilkinson I.B., McEniery C.M., Ahmadi-Abhari S., Singh-Manoux A., Kivimaki M., Brunner E.J. (2022). Aortic Pulse Wave Velocity as Adjunct Risk Marker for Assessing Cardiovascular Disease Risk: Prospective Study. Hypertension.

[4] Vlachopoulos C., Aznaouridis K., Stefanadis C. (2010). Prediction of cardiovascular events and all-cause mortality with arterial stiffness: A systematic review and meta-analysis. J. Am. Coll. Cardiol..

[5] Morgan E.E., Casabianca A.B., Khouri S.J., Kalinoski A.L. (2014). In vivo assessment of arterial stiffness in the isoflurane anesthetized spontaneously hypertensive rat. Cardiovasc. Ultrasound.

[6] Lindesay G., Ragonnet C., Chimenti S., Villeneuve N., Vayssettes-Courchay C. (2016). Age and hypertension strongly induce aortic stiffening in rats at basal and matched blood pressure levels. Physiol. Rep..

[7] Lindesay G., Bezie Y., Ragonnet C., Duchatelle V., Dharmasena C., Villeneuve N., Vayssettes-Courchay C. (2018). Differential Stiffening between the Abdominal and Thoracic Aorta: Effect of Salt Loading in Stroke-Prone Hypertensive Rats. J. Vasc. Res..

[8] Fabricio M.F., Jordao M.T., Miotto D.S., Ruiz T.F.R., Vicentini C.A., Lacchini S., Santos C.F., Michelini L.C., Amaral S.L. (2020). Standardization of a new non-invasive device for assessment of arterial stiffness in rats: Correlation with age-related arteries’ structure. MethodsX.

[9] Steppan J., Jandu S., Savage W., Wang H., Kang S., Narayanan R., Nyhan D., Santhanam L. (2020). Restoring Blood Pressure in Hypertensive Mice Fails to Fully Reverse Vascular Stiffness. Front. Physiol..

[10] Pilote L., Abrahamowicz M., Eisenberg M., Humphries K., Behlouli H., Tu J.V. (2008). Effect of different angiotensin-converting-enzyme inhibitors on mortality among elderly patients with congestive heart failure. CMAJ.

[11] Dinicolantonio J.J., Lavie C.J., O’Keefe J.H. (2013). Not all angiotensin-converting enzyme inhibitors are equal: Focus on ramipril and perindopril. Postgrad. Med..

[12] Ahimastos A.A., Aggarwal A., D’Orsa K.M., Formosa M.F., White A.J., Savarirayan R., Dart A.M., Kingwell B.A. (2007). Effect of perindopril on large artery stiffness and aortic root diameter in patients with Marfan syndrome: A randomized controlled trial. JAMA.

[13] Laurent S., Agabiti-Rosei C., Bruno R.M., Rizzoni D. (2022). Microcirculation and Macrocirculation in Hypertension: A Dangerous Cross-Link?. Hypertension.

[14] Sonawane K.B., Deshmukh A.A., Segal M.S. (2018). Pulse pressure, arterial stiffening, and the efficacy of renin-angiotensin system inhibitor combinations. J. Hum. Hypertens..

[15] Miotto D.S., Dionizio A., Jacomini A.M., Zago A.S., Buzalaf M.A.R., Amaral S.L. (2021). Identification of Aortic Proteins Involved in Arterial Stiffness in Spontaneously Hypertensive Rats Treated With Perindopril:A Proteomic Approach. Front. Physiol..

[16] Lopes S., Mesquita-Bastos J., Garcia C., Leitao C., Bertoquini S., Ribau V., Carvalho P., Oliveira J., Viana J., Figueiredo D. (2021). Physical Activity is Associated with Lower Arterial Stiffness in Patients with Resistant Hypertension. Heart Lung Circ..

[17] Miotto D.S., Duchatsch F., Macedo A.G., Ruiz T.F.R., Vicentini C.A., Amaral S.L. (2021). Perindopril Reduces Arterial Pressure and Does Not Inhibit Exercise-Induced Angiogenesis in Spontaneously Hypertensive Rats. J. Cardiovasc. Pharmacol..

[18] Zhou H., Wang S., Zhao C., He H. (2022). Effect of exercise on vascular function in hypertension patients: A meta-analysis of randomized controlled trials. Front. Cardiovasc. Med..

[19] Kohn J.C., Bordeleau F., Miller J., Watkins H.C., Modi S., Ma J., Azar J., Putnam D., Reinhart-King C.A. (2018). Beneficial Effects of Exercise on Subendothelial Matrix Stiffness are Short-Lived. J. Biomech. Eng..

[20] Herrera N.A., Jesus I., Dionisio E.J., Dionisio T.J., Santos C.F., Amaral S.L. (2017). Exercise Training Prevents Dexamethasone-induced Rarefaction. J. Cardiovasc. Pharmacol..

[21] Amaral S.L., Zorn T.M., Michelini L.C. (2000). Exercise training normalizes wall-to-lumen ratio of the gracilis muscle arterioles and reduces pressure in spontaneously hypertensive rats. J. Hypertens..

[22] Duchatsch F., Constantino P.B., Herrera N.A., Fabricio M.F., Tardelli L.P., Martuscelli A.M., Dionisio T.J., Santos C.F., Amaral S.L. (2018). Short-term exposure to dexamethasone promotes autonomic imbalance to the heart before hypertension. J. Am. Soc. Hypertens..

[23] Bradford M.M. (1976). A rapid and sensitive method for the quantitation of microgram quantities of protein utilizing the principle of protein-dye binding. Anal. Biochem..

[24] Batista T.B.D., Chaiben C.L., Penteado C.A.S., Nascimento J.M.C., Ventura T.M.O., Dionizio A., Rosa E.A.R., Buzalaf M.A.R., Azevedo-Alanis L.R. (2019). Salivary proteome characterization of alcohol and tobacco dependents. Drug Alcohol Depend..

[25] Dionizio A., Melo C.G.S., Sabino-Arias I.T., Araujo T.T., Ventura T.M.O., Leite A.L., Souza S.R.G., Santos E.X., Heubel A.D., Souza J.G. (2020). Effects of acute fluoride exposure on the jejunum and ileum of rats: Insights from proteomic and enteric innervation analysis. Sci. Total Environ..

[26] Leite A.L., Lobo J.G.V.M., Pereira H.A.B.S., Fernandes M.S., Martini T., Zucki F., Sumida D.H., Rigalli A., Buzalaf M.A. (2014). Proteomic analysis of gastrocnemius muscle in rats with streptozotocin-induced diabetes and chronically exposed to fluoride. PLoS ONE.

[27] Vasan R.S., Pan S., Xanthakis V., Beiser A., Larson M.G., Seshadri S., Mitchell G.F. (2022). Arterial Stiffness and Long-Term Risk of Health Outcomes: The Framingham Heart Study. Hypertension.

[28] Sang D.S., Zhang Q., Song D., Tao J., Wu S.L., Li Y.J. (2022). Association between brachial-ankle pulse wave velocity and cardiovascular and cerebrovascular disease in different age groups. Clin. Cardiol..

[29] Agbaje A.O. (2022). To prevent hypertension in Africans: Do we need to eat more vegetables?. Eur. J. Prev. Cardiol..

[30] Oh Y.S. (2018). Arterial stiffness and hypertension. Clin. Hypertens..

[31] Loboz-Rudnicka M., Jaroch J., Kruszynska E., Bociaga Z., Rzyczkowska B., Dudek K., Szuba A., Loboz-Grudzien K. (2018). Gender-related differences in the progression of carotid stiffness with age and in the influence of risk factors on carotid stiffness. Clin. Interv. Aging.

[32] Tardelli L.P., Duchatsch F., Herrera N.A., Vicentini C.A., Okoshi K., Amaral S.L. (2021). Differential effects of dexamethasone on arterial stiffness, myocardial remodeling and blood pressure between normotensive and spontaneously hypertensive rats. J. Appl. Toxicol..

[33] Tardelli L.P., Duchatsch F., Herrera N.A., Ruiz T.F.R., Pagan L.U., Vicentini C.A., Okoshi K., Amaral S.L. (2022). Benefits of combined exercise training on arterial stiffness and blood pressure in spontaneously hypertensive rats treated or not with dexamethasone. Front. Physiol..

[34] Barroso W.K.S., Rodrigues C.I.S., Bortolotto L.A., Mota-Gomes M.A., Brandao A.A., Feitosa A.D.M., Machado C.A., Poli-de-Figueiredo C.E., Amodeo C., Mion Junior D. (2021). Brazilian Guidelines of Hypertension—2020. Arq. Bras. De Cardiol..

[35] Pelliccia A., Sharma S., Gati S., Back M., Borjesson M., Caselli S., Collet J.P., Corrado D., Drezner J.A., Halle M. (2021). 2020 ESC Guidelines on sports cardiology and exercise in patients with cardiovascular disease. Eur. Heart J..

[36] Bull F.C., Al-Ansari S.S., Biddle S., Borodulin K., Buman M.P., Cardon G., Carty C., Chaput J.P., Chastin S., Chou R. (2020). World Health Organization 2020 guidelines on physical activity and sedentary behaviour. Br. J. Sport. Med..

[37] Vlachopoulos C., Terentes-Printzios D., Laurent S., Nilsson P.M., Protogerou A.D., Aznaouridis K., Xaplanteris P., Koutagiar I., Tomiyama H., Yamashina A. (2019). Association of Estimated Pulse Wave Velocity with Survival: A Secondary Analysis of SPRINT. JAMA Netw. Open.

[38] Ong K.T., Delerme S., Pannier B., Safar M.E., Benetos A., Laurent S., Boutouyrie P., Investigators (2011). Aortic stiffness is reduced beyond blood pressure lowering by short-term and long-term antihypertensive treatment: A meta-analysis of individual data in 294 patients. J. Hypertens..

[39] Ferrari R., Pasanisi G., Notarstefano P., Campo G., Gardini E., Ceconi C. (2005). Specific properties and effect of perindopril in controlling the renin-angiotensin system. Am. J. Hypertens..

[40] Amaral S.L., Michelini L.C. (2011). Effect of gender on training-induced vascular remodeling in SHR. Braz. J. Med. Biol. Res..

[41] Fernandes T., Magalhaes F.C., Roque F.R., Phillips M.I., Oliveira E.M. (2012). Exercise training prevents the microvascular rarefaction in hypertension balancing angiogenic and apoptotic factors: Role of microRNAs-16, -21, and -126. Hypertension.

[42] Herrera N.A., Jesus I., Shinohara A.L., Dionisio T.J., Santos C.F., Amaral S.L. (2016). Exercise training attenuates dexamethasone-induced hypertension by improving autonomic balance to the heart, sympathetic vascular modulation and skeletal muscle microcirculation. J. Hypertens..

[43] Constantino P.B., Dionisio T.J., Duchatsch F., Herrera N.A., Duarte J.O., Santos C.F., Crestani C.C., Amaral S.L. (2017). Exercise attenuates dexamethasone-induced hypertension through an improvement of baroreflex activity independently of the renin-angiotensin system. Steroids.

[44] Lopes S., Afreixo V., Teixeira M., Garcia C., Leitao C., Gouveia M., Figueiredo D., Alves A.J., Polonia J., Oliveira J. (2021). Exercise training reduces arterial stiffness in adults with hypertension: A systematic review and meta-analysis. J. Hypertens..

[45] Xin C., Ye M., Zhang Q., He H. (2022). Effect of Exercise on Vascular Function and Blood Lipids in Postmenopausal Women: A Systematic Review and Network Meta-Analysis. Int. J. Environ. Res. Public Health.

[46] Saz-Lara A., Cavero-Redondo I., Alvarez-Bueno C., Notario-Pacheco B., Reina-Gutierrez S., Sequi-Dominguez I., Ruiz J.R., Martinez-Vizcaino V. (2021). What type of physical exercise should be recommended for improving arterial stiffness on adult population? A network meta-analysis. Eur. J. Cardiovasc. Nurs..

[47] Li G., Lv Y., Su Q., You Q., Yu L. (2022). The effect of aerobic exercise on pulse wave velocity in middle-aged and elderly people: A systematic review and meta-analysis of randomized controlled trials. Front. Cardiovasc. Med..

[48] Mynard J.P., Clarke M.M. (2019). Arterial Stiffness, Exercise Capacity and Cardiovascular Risk. Heart Lung Circ..

[49] Son W.M., Sung K.D., Bharath L.P., Choi K.J., Park S.Y. (2017). Combined exercise training reduces blood pressure, arterial stiffness, and insulin resistance in obese prehypertensive adolescent girls. Clin. Exp. Hypertens..

[50] Otsuki T., Namatame H., Yoshikawa T., Zempo-Miyaki A. (2020). Combined aerobic and low-intensity resistance exercise training increases basal nitric oxide production and decreases arterial stiffness in healthy older adults. J. Clin. Biochem. Nutr..

[51] Jordao M.T., Ladd F.V., Coppi A.A., Chopard R.P., Michelini L.C. (2011). Exercise training restores hypertension-induced changes in the elastic tissue of the thoracic aorta. J. Vasc. Res..

[52] Morales-Quinones M., Ramirez-Perez F.I., Foote C.A., Ghiarone T., Ferreira-Santos L., Bloksgaard M., Spencer N., Kimchi E.T., Manrique-Acevedo C., Padilla J. (2020). LIMK (LIM Kinase) Inhibition Prevents Vasoconstriction- and Hypertension-Induced Arterial Stiffening and Remodeling. Hypertension.

[53] Simone L.C., Naslavsky N., Caplan S. (2014). Scratching the surface: Actin’ and other roles for the C-terminal Eps15 homology domain protein, EHD2. Histol. Histopathol..

[54] Matthaeus C., Lian X., Kunz S., Lehmann M., Zhong C., Bernert C., Lahmann I., Muller D.N., Gollasch M., Daumke O. (2019). eNOS-NO-induced small blood vessel relaxation requires EHD2-dependent caveolae stabilization. PLoS ONE.

[55] Torrino S., Shen W.W., Blouin C.M., Mani S.K., Viaris de Lesegno C., Bost P., Grassart A., Koster D., Valades-Cruz C.A., Chambon V. (2018). EHD2 is a mechanotransducer connecting caveolae dynamics with gene transcription. J. Cell Biol..

[56] Lacolley P., Regnault V., Segers P., Laurent S. (2017). Vascular Smooth Muscle Cells and Arterial Stiffening: Relevance in Development, Aging, and Disease. Physiol. Rev..

[57] Goel S.A., Guo L.W., Shi X.D., Kundi R., Sovinski G., Seedial S., Liu B., Kent K.C. (2013). Preferential secretion of collagen type 3 versus type 1 from adventitial fibroblasts stimulated by TGF-beta/Smad3-treated medial smooth muscle cells. Cell Signal.

[58] Wittig C., Szulcek R. (2021). Extracellular Matrix Protein Ratios in the Human Heart and Vessels: How to Distinguish Pathological from Physiological Changes?. Front. Physiol..

[59] de Paula V.F., Tardelli L.P., Amaral S.L. (2023). Dexamethasone-Induced Arterial Stiffening Is Attenuated by Training due to a Better Balance Between Aortic Collagen and Elastin Levels. Cardiovasc. Drugs Ther..

[60] Guers J.J., Farquhar W.B., Edwards D.G., Lennon S.L. (2019). Voluntary Wheel Running Attenuates Salt-Induced Vascular Stiffness Independent of Blood Pressure. Am. J. Hypertens..

[61] Dab H., Kacem K., Hachani R., Dhaouadi N., Hodroj W., Sakly M., Randon J., Bricca G. (2012). Physiological regulation of extracellular matrix collagen and elastin in the arterial wall of rats by noradrenergic tone and angiotensin II. J. Renin-Angiotensin-Aldosterone Syst..

[62] He F., Chu J.F., Chen H.W., Lin W., Lin S., Chen Y.Q., Peng J., Chen K.J. (2020). Qingxuan Jiangya Decoction (清眩降压汤) Prevents Blood Pressure Elevation and Ameliorates Vascular Structural Remodeling via Modulating TGF-beta 1/Smad Pathway in Spontaneously Hypertensive Rats. Chin. J. Integr. Med..

[63] Fassot C., Briet M., Rostagno P., Barbry P., Perret C., Laude D., Boutouyrie P., Bozec E., Bruneval P., Latremouille C. (2008). Accelerated arterial stiffening and gene expression profile of the aorta in patients with coronary artery disease. J. Hypertens..

[64] Deng T., Liu Y., Gael A., Fu X., Deng X., Liu Y., Wu Y., Wu Y., Wang H., Deng Y. (2022). Study on Proteomics-Based Aortic Dissection Molecular Markers Using iTRAQ Combined with Label Free Techniques. Front. Physiol..

