# Supplementary material for: Physical Training vs. Perindopril Treatment on Arterial Stiffening of Spontaneously Hypertensive Rats: A Proteomic Analysis and Possible Mechanisms
Source: Biomedicines. 2023 May 6;11(5):1381. doi: 10.3390/biomedicines11051381 (PMC10216057; doi:10.3390/biomedicines11051381)
Supplement: Supplementary file 1 [file biomedicines-11-01381-s001.zip › biomedicines-2339110-supplementary.pdf]

Supplementary Material

Table S1: Protein analysis in ClueGO plugins and the number of genes involved within the process biologic category between SHR$_P$ vs. SHR$_C$ groups.

Process Biologic

Term	Nr. Genes
cellular response to epidermal growth factor stimulus	3.00
collagen fibril organization	3.00
structural constituent of cytoskeleton	22.00
mitochondrial electron transport. cytochrome c to oxygen	2.00
protein homotetramerization	3.00
cellular response to interleukin-4	4.00
cerebellar cortex development	4.00
regulation of mitotic spindle organization	3.00
acute-phase response	5.00
stress response to metal ion	2.00
positive regulation of axon guidance	2.00
endothelial cell chemotaxis	2.00
dendritic cell chemotaxis	3.00
glial cell proliferation	7.00
hydrogen peroxide metabolic process	5.00
platelet activation	6.00
chondroitin sulfate proteoglycan metabolic process	2.00
regulation of transepithelial transport	2.00
response to lead ion	4.00
sarcomere organization	6.00
mesenchyme migration	4.00
regulation of protein dephosphorylation	9.00
establishment or maintenance of epithelial cell apical/basal polarity	4.00
regulation of cholesterol metabolic process	2.00
lactate metabolic process	3.00
response to hydrogen peroxide	16.00
regulation of oxidative stress-induced intrinsic apoptotic signaling pathway	5.00
ventricular system development	2.00
energy derivation by oxidation of organic compounds	19.00
intermediate filament organization	11.00
positive regulation of blood vessel endothelial cell migration	5.00
regulation of calcium-mediated signaling	5.00
thioester biosynthetic process	2.00
structural constituent of synapse	7.00
cellular response to reactive oxygen species	11.00
regulation of NIK/NF-kappaB signaling	6.00
actin filament fragmentation	2.00
response to platinum ion	2.00
negative regulation of extrinsic apoptotic signaling pathway	6.00
ribonucleoside metabolic process	2.00
cellular response to cadmium ion	2.00

positive regulation of viral process	4.00
cellular oxidant detoxification	9.00
regulation of response to oxidative stress	10.00
negative regulation of vascular associated smooth muscle cell proliferation	6.00
ATP-dependent protein folding chaperone	8.00
positive regulation of epithelial cell migration	9.00
purine-containing compound biosynthetic process	11.00
chaperone-mediated protein folding	8.00
negative regulation of striated muscle cell apoptotic process	2.00
regulation of wound healing	7.00
purine ribonucleoside triphosphate biosynthetic process	7.00
structural constituent of postsynapse	5.00
supramolecular fiber organization	44.00
peroxidase activity	6.00
fatty acid beta-oxidation	7.00
regulation of polysaccharide biosynthetic process	2.00
structural constituent of postsynaptic intermediate filament cytoskeleton	2.00
intermediate filament cytoskeleton organization	11.00
pyrimidine nucleoside triphosphate biosynthetic process	2.00
negative regulation of exocytosis	4.00
male meiosis I	4.00
blood vessel endothelial cell migration	7.00
dopamine receptor signaling pathway	5.00
response to reactive oxygen species	20.00
regulation of vascular associated smooth muscle cell proliferation	9.00
response to inorganic substance	43.00

Table S2: Protein analysis in ClueGO plug-ins and the number of genes involved within the process biological category between SHR_T vs SHR_C groups.

Process Biological

Term	Nr. Genes
collagen fibril organization	4.00
regulation of cholesterol metabolic process	3.00
cellular response to epidermal growth factor stimulus	4.00
tricarboxylic acid metabolic process	2.00
negative regulation of blood vessel endothelial cell migration	2.00
acute-phase response	6.00
fatty acid beta-oxidation using acyl-CoA dehydrogenase	3.00
regulation of protein dephosphorylation	5.00
positive regulation of bone resorption	2.00
response to lead ion	4.00
cardiac epithelial to mesenchymal transition	2.00
sarcomere organization	4.00
actomyosin structure organization	11.00
response to heat	10.00
cellular oxidant detoxification	7.00
regulation of reactive oxygen species metabolic process	9.00
nucleus localization	2.00
hydrogen peroxide metabolic process	5.00
superoxide metabolic process	6.00
positive regulation of gliogenesis	5.00
ATP-dependent protein folding chaperone	7.00
response to ATP	2.00
negative regulation of extrinsic apoptotic signaling pathway	6.00
cellular response to fatty acid	5.00
positive regulation of sterol transport	2.00
mesenchyme migration	4.00
myofibril assembly	8.00
protein refolding	5.00
gluconeogenesis	5.00
face morphogenesis	2.00
regulation of superoxide metabolic process	4.00
negative regulation of ion transmembrane transport	7.00
collagen biosynthetic process	4.00
cerebellar Purkinje cell layer development	4.00
negative regulation of striated muscle cell apoptotic process	2.00
structural constituent of synapse	4.00
fibrinolysis	3.00
negative regulation of hyaluronan biosynthetic process	2.00
chaperone cofactor-dependent protein refolding	5.00
protein localization to chromatin	2.00
positive regulation of lamellipodium organization	2.00
cellular response to butyrate	2.00
regulation of dopamine biosynthetic process	2.00

regulation of wound healing	8.00
regulation of cellular response to oxidative stress	7.00
actin filament fragmentation	3.00
protein folding chaperone	8.00
regulation of response to oxidative stress	8.00

Table S3. The protein of comparison SHR_P vs. SHR_C. 138 proteins were identified, with 73 *upregulated* and 2 *downregulated* significantly in the first group of the comparison and unique from each group with p<0.05**.

ccession [a]	*Description*	*Score*	*SHR_p:SHR_c_ Ratio* [b]	*SHR_p:SHR_c __P* [c]	*Up / intermediate / Down* [d]
Q9ER34	Aconitate hydratase_ mitochondrial	83	1.67	< 0.01	↑
P68035	Actin_ alpha cardiac muscle 1	5542	1.07	< 0.01	↑
P68136	Actin_ alpha skeletal muscle	5242	1.07	< 0.01	↑
P62738	Actin_ aortic smooth muscle	5534	1.07	< 0.01	↑
P60711	Actin_ cytoplasmic 1	4354	1.12	< 0.01	↑
P63259	Actin_ cytoplasmic 2	4358	1.12	< 0.01	↑
P63269	Actin_ gamma-enteric smooth muscle	5534	1.07	< 0.01	↑
Q9Z1P2	Alpha-actinin-1	719	1.30	< 0.01	↑
Q9QXQ0	Alpha-actinin-4	431	1.31	< 0.01	↑
Q07936	Annexin A2	474	1.38	< 0.01	↑
P15999	ATP synthase subunit alpha_ mitochondrial	175	1.54	< 0.01	↑
P10719	ATP synthase subunit beta_ mitochondrial	87	1.38	< 0.01	↑
P47853	Biglycan	227	1.72	< 0.01	↑
P02454	Collagen alpha-1(I) chain	47	1.88	< 0.01	↑
P47875	Cysteine and glycine-rich protein 1	1148	1.45	< 0.01	↑
P36201	Cysteine-rich protein 2	176	1.63	< 0.01	↑
P48675	Desmin	467	1.26	< 0.01	↑
Q4V8H8	EH domain-containing protein 2	225	1.45	< 0.01	↑
P62630	Elongation factor 1-alpha 1	298	1.62	< 0.01	↑
P70623	Fatty acid-binding protein_ adipocyte	436	1.60	< 0.01	↑
P06399	Fibrinogen alpha chain	112	1.32	< 0.01	↑
Q9WVH8	Fibulin-5	52	1.32	< 0.01	↑
P11762	Galectin-1	260	1.75	< 0.01	↑
P08010	Glutathione S-transferase Mu 2	217	1.58	< 0.01	↑
P0DMW0	Heat shock 70 kDa protein 1A	849	1.28	< 0.01	↑
P55063	Heat shock 70 kDa protein 1-like	597	1.31	< 0.01	↑
P63018	Heat shock cognate 71 kDa protein	852	1.36	< 0.01	↑
P42930	Heat shock protein beta-1	848	1.54	< 0.01	↑
P14659	Heat shock-related 70 kDa protein 2	691	1.39	< 0.01	↑
Q6IG12	Keratin_ type II cytoskeletal 7	193	1.67	< 0.01	↑
Q10758	Keratin_ type II cytoskeletal 8	193	1.70	< 0.01	↑
P18666	Myosin regulatory light chain 12B	975	1.27	< 0.01	↑
Q64122	Myosin regulatory light polypeptide 9	4053	1.34	< 0.01	↑
Q9JLT0	Myosin-10	71	1.49	< 0.01	↑
Q62812	Myosin-9	68	1.52	< 0.01	↑
P10111	Peptidyl-prolyl cis-trans isomerase A	1750	1.42	< 0.01	↑
P62963	Profilin-1	3064	1.19	< 0.01	↑
P02770	Serum albumin	7160	1.09	< 0.01	↑
P31232	Transgelin	9127	1.40	< 0.01	↑
P68370	Tubulin alpha-1A chain	237	1.67	< 0.01	↑
Q6P9V9	Tubulin alpha-1B chain	237	1.67	< 0.01	↑
Q6AYZ1	Tubulin alpha-1C chain	237	1.68	< 0.01	↑
Q68FR8	Tubulin alpha-3 chain	111	1.84	< 0.01	↑

Q5XIF6	Tubulin alpha-4A chain	107	1.73	< 0.01	↑
Q6AY56	**Tubulin alpha-8 chain**	**46**	**2.20**	**< 0.01**	↑
P85108	Tubulin beta-2A chain	94	1.49	< 0.01	↑
Q3KRE8	Tubulin beta-2B chain	94	1.49	< 0.01	↑
Q4QRB4	Tubulin beta-3 chain	94	1.48	< 0.01	↑
Q6P9T8	Tubulin beta-4B chain	467	1.48	< 0.01	↑
P69897	Tubulin beta-5 chain	94	1.49	< 0.01	↑
P31000	Vimentin	1733	1.23	< 0.01	↑
Q5RKI0	WD repeat-containing protein 1	192	1.32	< 0.01	↑
*P07150 **	Annexin A1	94	1.52	0.01	↑
P85125	Caveolae-associated protein 1	150	1.51	0.01	↑
P06761	Endoplasmic reticulum chaperone BiP	248	1.22	0.01	↑
P0DMW1	Heat shock 70 kDa protein 1B	845	1.30	0.01	↑
Q00715	Histone H2B type 1	122	1.48	0.01	↑
P15650	Long-chain specific acyl-CoA dehydrogenase_ mitochondrial	93	1.51	0.01	↑
P02600	Myosin light chain 1/3_ skeletal muscle isoform	809	1.28	0.01	↑
P16409	Myosin light chain 3	787	1.30	0.01	↑
P13832	Myosin regulatory light chain RLC-A	975	1.26	0.01	↑
P85973	Purine nucleoside phosphorylase	155	1.31	0.01	↑
Q7M0E3	Destrin	1706	1.22	0.02	↑
P20760	Ig gamma-2A chain C region	643	1.13	0.02	↑
Q62736	Non-muscle caldesmon	274	1.72	0.02	↑
P21807	Peripherin	195	1.35	0.02	↑
P12346	Serotransferrin	1002	1.14	0.02	↑
P63102	14-3-3 protein zeta/delta	142	1.34	0.03	↑
Q01129	Decorin	150	1.55	0.03	↑
P05065	Fructose-bisphosphate aldolase A	202	1.26	0.03	↑
Q6P6Q2	Keratin_ type II cytoskeletal 5	76	1.70	0.03	↑
P04636	Malate dehydrogenase_ mitochondrial	346	1.40	0.03	↑
Q5XI73	Rho GDP-dissociation inhibitor 1	327	1.36	0.04	↑
*P14668 **	Annexin A5	145	1.25	0.95	----
P04797	Glyceraldehyde-3-phosphate dehydrogenase	1896	1.13	0.95	----
A6YP92	Homeobox protein ARX	33	1.75	0.95	----
P11598	Protein disulfide-isomerase A3	100	1.42	0.95	----
P16636	Protein-lysine 6-oxidase	251	1.46	0.95	----
P50398	Rab GDP dissociation inhibitor alpha	112	1.79	0.95	----
Q64119	Myosin light polypeptide 6	13395	1.09	0.94	----
P25113	Phosphoglycerate mutase 1	276	1.55	0.94	----
P50137	Transketolase	50	1.40	0.94	----
P48037	Annexin A6	120	1.15	0.93	----
P34058	Heat shock protein HSP 90-beta	106	1.67	0.93	----
P23565	Alpha-internexin	69	1.31	0.90	----
P62632	Elongation factor 1-alpha 2	51	2.03	0.90	----
P02680	Fibrinogen gamma chain	682	1.19	0.90	----
P20761	Ig gamma-2B chain C region	130	1.19	0.90	----
P04692	Tropomyosin alpha-1 chain	736	1.19	0.90	----
P68511	14-3-3 protein eta	63	1.34	0.89	----
O88989	Malate dehydrogenase_ cytoplasmic	103	1.34	0.89	----
P11506	Plasma membrane calcium-transporting ATPase 2	71	1.40	0.89	----

P07943	Aldose reductase	254	1.17	0.88	----
Q68FY0	Cytochrome b-c1 complex subunit 1_ mitochondrial	160	1.35	0.87	----
P70490	Lactadherin	112	1.27	0.87	----
P58775	Tropomyosin beta chain	736	1.17	0.87	----
Q4FZU2	Keratin_ type II cytoskeletal 6A	76	1.54	0.86	----
Q08163	Adenylyl cyclase-associated protein 1	165	1.15	0.85	----
P16617	Phosphoglycerate kinase 1	73	1.55	0.84	----
P11980	Pyruvate kinase PKM	40	1.51	0.83	----
Q6IE24	Inactive ubiquitin carboxyl-terminal hydrolase 54	36	1.13	0.82	----
P50399	Rab GDP dissociation inhibitor beta	52	1.65	0.81	----
P09495	Tropomyosin alpha-4 chain	50	1.22	0.81	----
Q6IG05	Keratin_ type II cytoskeletal 75	76	1.45	0.79	----
P47819	Glial fibrillary acidic protein	52	1.45	0.78	----
P01836	Ig kappa chain C region_ A allele	701	1.22	0.78	----
Q6IG00	Keratin_ type II cytoskeletal 4	52	1.49	0.77	----
P09117	Fructose-bisphosphate aldolase C	79	1.51	0.74	----
Q63610	Tropomyosin alpha-3 chain	50	1.17	0.74	----
P15800	Laminin subunit beta-2	47	1.22	0.73	----
P24090	Alpha-2-HS-glycoprotein	275	1.15	0.72	----
P14480	Fibrinogen beta chain	941	1.08	0.72	----
P51886	Lumican	1267	1.00	0.72	----
P20759	Ig gamma-1 chain C region	31	1.39	0.70	----
P01026	Complement C3	57	1.12	0.69	----
P13437	3-ketoacyl-CoA thiolase_ mitochondrial	85	1.16	0.67	----
P45592	Cofilin-1	297	1.06	0.67	----
P61983	14-3-3 protein gamma	53	1.09	0.65	----
P07632	Superoxide dismutase [Cu-Zn]	125	1.07	0.65	----
P04764	Alpha-enolase	138	1.05	0.62	----
P62260	14-3-3 protein epsilon	148	1.09	0.59	----
P11884	Aldehyde dehydrogenase_ mitochondrial	90	1.05	0.59	----
P20059	Hemopexin	409	1.02	0.58	----
P68255	14-3-3 protein theta	164	1.08	0.53	----
P35213	14-3-3 protein beta/alpha	164	1.03	0.43	----
P62982	Ubiquitin-40S ribosomal protein S27a	274	0.93	0.42	----
P04906	Glutathione S-transferase P	114	0.95	0.39	----
Q66HD0	Endoplasmin	51	0.90	0.37	----
Q63429	Polyubiquitin-C	274	0.92	0.35	----
P12839	Neurofilament medium polypeptide	79	0.92	0.34	----
P0CG51	Polyubiquitin-B	274	0.90	0.33	----
Q63598	Plastin-3	90	0.86	0.32	----
P62986	Ubiquitin-60S ribosomal protein L40	274	0.90	0.29	----
Q68FU3	Electron transfer flavoprotein subunit beta	536	0.68	0.19	----
P06866	Haptoglobin	335	0.88	0.17	----
P11517	Hemoglobin subunit beta-2	3388	0.86	0.06	----
P01946	Hemoglobin subunit alpha-1/2	14931	0.62	< 0.01	↓
P02091	Hemoglobin subunit beta-1	10419	0.76	< 0.01	↓
P26772	10 kDa heat shock protein_ mitochondrial	416			SHR_P
Q5XI78	2-oxoglutarate dehydrogenase_ mitochondrial	90			SHR_P
P17764	Acetyl-CoA acetyltransferase_ mitochondrial	180			SHR_P

Q5XIK1	Actin-related protein T1	73			SHR$_P$
P11030	Acyl-CoA-binding protein	271			SHR$_P$
P17475	Alpha-1-antiproteinase	149			SHR$_P$
P10759	AMP deaminase 1	101			SHR$_P$
Q5M9H0	Ankyrin repeat and SAM domain-containing protein 3	98			SHR$_P$
D3ZAF6	ATP synthase subunit f_ mitochondrial	55			SHR$_P$
Q925T8	BMP/retinoic acid-inducible neural-specific protein 1	95			SHR$_P$
P0DP29	Calmodulin-1	145			SHR$_P$
P0DP30	Calmodulin-2	145			SHR$_P$
P0DP31	Calmodulin-3	145			SHR$_P$
Q08290	Calponin-1	92			SHR$_P$
P18418	Calreticulin	109			SHR$_P$
P24268	Cathepsin D	117			SHR$_P$
Q5U3Z0	Cilia- and flagella-associated protein 298	158			SHR$_P$
P11442	Clathrin heavy chain 1	71			SHR$_P$
P05371	Clusterin	93			SHR$_P$
Q4KM47	Cyclin-dependent kinase 10	102			SHR$_P$
P14841	Cystatin-C	264			SHR$_P$
P11240	Cytochrome c oxidase subunit 5A_ mitochondrial	587			SHR$_P$
P10818	Cytochrome c oxidase subunit 6A1_ mitochondrial	631			SHR$_P$
Q62871	Cytoplasmic dynein 1 intermediate chain 2	109			SHR$_P$
Q5BK18	Cytosolic iron-sulfur assembly component 3	92			SHR$_P$
Q8K5A9	Death domain-containing membrane protein NRADD	70			SHR$_P$
P06214	Delta-aminolevulinic acid dehydratase	75			SHR$_P$
Q62952	Dihydropyrimidinase-related protein 3	103			SHR$_P$
Q62967	Diphosphomevalonate decarboxylase	95			SHR$_P$
Q66HC9	Dynein intermediate chain 2_ axonemal	95			SHR$_P$
Q00911	Early growth response protein 4	140			SHR$_P$
P13803	Electron transfer flavoprotein subunit alpha_ mitochondrial	189			SHR$_P$
P05197	Elongation factor 2	108			SHR$_P$
P14604	Enoyl-CoA hydratase_ mitochondrial	46			SHR$_P$
Q5RKI1	Eukaryotic initiation factor 4A-II	92			SHR$_P$
P50609	Fibromodulin	125			SHR$_P$
Q9WUH4	Four and a half LIM domains protein 1	413			SHR$_P$
P10860	Glutamate dehydrogenase 1_ mitochondrial	253			SHR$_P$
Q99MZ4	Glutathione hydrolase 7	121			SHR$_P$
Q63406	Guanine nucleotide exchange factor DBS	61			SHR$_P$
P10824	Guanine nucleotide-binding protein G(i) subunit alpha-1	104			SHR$_P$
P04897	Guanine nucleotide-binding protein G(i) subunit alpha-2	157			SHR$_P$
P08753	Guanine nucleotide-binding protein G(k) subunit alpha	104			SHR$_P$
P59215	Guanine nucleotide-binding protein G(o) subunit alpha	104			SHR$_P$
P38406	Guanine nucleotide-binding protein G(olf) subunit alpha	104			SHR$_P$

P63095	Guanine nucleotide-binding protein G(s) subunit alpha isoforms short	104			SHR$_P$
Q63803	Guanine nucleotide-binding protein G(s) subunit alpha isoforms XLas	104			SHR$_P$
P29348	Guanine nucleotide-binding protein G(t) subunit alpha-3	104			SHR$_P$
Q63210	Guanine nucleotide-binding protein subunit alpha-12	101			SHR$_P$
Q6Q7Y5	Guanine nucleotide-binding protein subunit alpha-13	101			SHR$_P$
Q9Z136	Hamartin	51			SHR$_P$
P62959	Histidine triad nucleotide-binding protein 1	223			SHR$_P$
Q99MK2	Histone acetyltransferase KAT5	156			SHR$_P$
Q00729	Histone H2B type 1-A	1001			SHR$_P$
Q9ESM2	Hyaluronan and proteoglycan link protein 2	59			SHR$_P$
P20762	Ig gamma-2C chain C region	127			SHR$_P$
P01835	Ig kappa chain C region_ B allele	2149			SHR$_P$
Q99NA5	Isocitrate dehydrogenase [NAD] subunit alpha_ mitochondrial	82			SHR$_P$
Q6IFW6	Keratin_ type I cytoskeletal 10	74			SHR$_P$
P25030	Keratin_ type I cytoskeletal 20	65			SHR$_P$
P70615	Lamin-B1	53			SHR$_P$
Q99MZ8	LIM and SH3 domain protein 1	97			SHR$_P$
Q5XI07	Lipoma-preferred partner homolog	326			SHR$_P$
A1A5P5	LisH domain-containing protein ARMC9	128			SHR$_P$
P04642	L-lactate dehydrogenase A chain	122			SHR$_P$
P42123	L-lactate dehydrogenase B chain	214			SHR$_P$
P30904	Macrophage migration inhibitory factor	169			SHR$_P$
P02761	Major urinary protein	48			SHR$_P$
O35763	Moesin	109			SHR$_P$
P16884	Neurofilament heavy polypeptide	96			SHR$_P$
P19527	Neurofilament light polypeptide	89			SHR$_P$
O08658	Nuclear pore complex protein Nup88	54			SHR$_P$
Q05982	Nucleoside diphosphate kinase A	480			SHR$_P$
P19804	Nucleoside diphosphate kinase B	447			SHR$_P$
Q63371	P2Y purinoceptor 6	95			SHR$_P$
P27657	Pancreatic triacylglycerol lipase	58			SHR$_P$
P52944	PDZ and LIM domain protein 1	213			SHR$_P$
Q66HS7	PDZ and LIM domain protein 3	128			SHR$_P$
Q62920	PDZ and LIM domain protein 5	93			SHR$_P$
Q9Z1Z9	PDZ and LIM domain protein 7	118			SHR$_P$
Q63716	Peroxiredoxin-1	105			SHR$_P$
Q9Z221	Polyamine-modulated factor 1-binding protein 1	106			SHR$_P$
D4ABH7	Pre-miRNA 5'-monophosphate methyltransferase	148			SHR$_P$
P04785	Protein disulfide-isomerase	91			SHR$_P$
Q9QZQ5	Protein NOV homolog	256			SHR$_P$
P05964	Protein S100-A6	1473			SHR$_P$
P98106	P-selectin	57			SHR$_P$
P49432	Pyruvate dehydrogenase E1 component subunit beta_ mitochondrial	53			SHR$_P$
P29315	Ribonuclease inhibitor	58			SHR$_P$

P57760	Serine/threonine-protein kinase 16	110			SHR_P
Q9QWN8	Spectrin beta chain_ non-erythrocytic 2	54			SHR_P
G3V7P1	Syntaxin-12	92			SHR_P
Q63635	Syntaxin-6	91			SHR_P
Q5XHX6	Thioredoxin domain-containing protein 2	55			SHR_P
Q6IE14	Transmembrane protease serine 11B-like protein	106			SHR_P
P02767	Transthyretin	216			SHR_P
P27435	Tryptase	591			SHR_P
Q9EQT5	Tubulointerstitial nephritis antigen-like	310			SHR_P
Q498R7	UPF0587 protein C1orf123 homolog	79			SHR_P
Q6AY86	Vacuolar protein sorting-associated protein 26A	104			SHR_P
O54975	Xaa-Pro aminopeptidase 1	107			SHR_P
P80299	Bifunctional epoxide hydrolase 2	47			SHR_C
Q4V8E4	Cilia- and flagella-associated protein 36	83			SHR_C
O55096	Dipeptidyl peptidase 3	45			SHR_C
Q91XQ4	DNA-directed RNA polymerase II subunit GRINL1A	69			SHR_C
Q62862	Dual specificity mitogen-activated protein kinase kinase 5	67			SHR_C
Q5EZ72	Ectonucleotide pyrophosphatase/phosphodiesterase family member 7	42			SHR_C
Q66H04	F-box only protein 43	68			SHR_C
P13255	Glycine N-methyltransferase	44			SHR_C
Q5XHZ0	Heat shock protein 75 kDa_ mitochondrial	47			SHR_C
P06762	Heme oxygenase 1	60			SHR_C
E9PU28	Inosine-5'-monophosphate dehydrogenase 2	79			SHR_C
Q99J82	Integrin-linked protein kinase	75			SHR_C
P18588	Interferon-induced GTP-binding protein Mx1	61			SHR_C
P56574	Isocitrate dehydrogenase [NADP]_ mitochondrial	74			SHR_C
Q5U2U7	mRNA cap guanine-N7 methyltransferase	99			SHR_C
A7E3N2	Neutrophil cytosol factor 2	52			SHR_C
O88767	Protein/nucleic acid deglycase DJ-1	229			SHR_C
Q9JK11	Reticulon-4	39			SHR_C
P42346	Serine/threonine-protein kinase mTOR	52			SHR_C
D3ZVU1	SprT-like domain-containing protein Spartan	72			SHR_C
P01048	T-kininogen 1	55			SHR_C

[a] identification of proteins in the Uniprot database (http://www.uniprot.org/).
[b] Significant difference in expression proteins up regulation, down regulation and intermediates.
[c] p value ($p<0.05$).
[d] Up regulated (↑), Intermediate (----, present in both comparison groups), down regulated (↓) and unique each comparison group (SHR_P and SHR_C).
**Adapted with permission from ref 15.

Table S4. The protein of comparison SHR_T vs. SHR_C. 123 proteins were identified, with 7 *upregulated* and 22 *downregulated* significantly in the first group of the comparison and unique from each group with p<0.05.

Accession [a]	*Description*	*Score*	*SHR_T: SHR_c_Ratio [b]*	*SHR_T: SHR_c __P [c]*	*Up / intermediate / Down / Unique*
P68035	Actin_ alpha cardiac muscle 1	5542	1.32	< 0.01	↑
P63269	Actin_ gamma-enteric smooth muscle	5534	1.32	< 0.01	↑
P47853	Biglycan	227	1.57	< 0.01	↑
P06761	Endoplasmic reticulum chaperone BiP	248	1.34	< 0.01	↑
P70490	**Lactadherin**	**112**	**3.16**	**< 0.01**	↑
Q6AY56	Tubulin alpha-8 chain	46	1.86	< 0.01	↑
Q9JLT0	Myosin-10	71	1.31	0.02	↑
P21807	Peripherin	195	1.38	0.95	----
Q6AYZ1	Tubulin alpha-1C chain	237	1.15	0.95	----
P01026	Complement C3	57	1.43	0.94	----
P48675	Desmin	467	1.13	0.94	----
P16636	Protein-lysine 6-oxidase V=2	251	1.42	0.94	----
Q68FR8	Tubulin alpha-3 chain	111	1.23	0.93	----
P58775	Tropomyosin beta chain	736	1.20	0.92	----
P06762	Heme oxygenase 1	60	1.54	0.89	----
Q62736	Non-muscle caldesmon	274	1.39	0.89	----
P68370	Tubulin alpha-1A chain	237	1.14	0.89	----
Q5XIF6	Tubulin alpha-4A chain	107	1.19	0.89	----
Q6P9V9	Tubulin alpha-1B chain	237	1.12	0.87	----
P01836	Ig kappa chain C region_ A allele	701	1.25	0.86	----
P23565	Alpha-internexin	69	1.20	0.85	----
P20761	Ig gamma-2B chain C region	130	1.19	0.85	----
Q63598	Plastin-3	90	1.52	0.85	----
P11762	Galectin-1	260	1.22	0.84	----
P07943	Aldose reductase OS=Rattus norvegicus	254	1.21	0.83	----
Q5XI73	Rho GDP-dissociation inhibitor 1	327	1.19	0.83	----
P04692	Tropomyosin alpha-1 chain	736	1.19	0.83	----
P12839	Neurofilament medium polypeptide	79	1.12	0.81	----
P14668	Annexin A5	145	1.15	0.80	----
P18666	Myosin regulatory light chain 12B	975	1.13	0.79	----
P13832	Myosin regulatory light chain RLC-A	975	1.11	0.78	----
Q6IG00	Keratin_ type II cytoskeletal 4	52	1.72	0.77	----
P04764	Alpha-enolase	138	1.16	0.75	----
P63102	14-3-3 protein zeta/delta	142	1.17	0.74	----
P07150	Annexin A1	94	1.20	0.74	----
P11598	Protein disulfide-isomerase A3	100	1.22	0.74	----
O88989	Malate dehydrogenase_ cytoplasmic	103	1.23	0.73	----
P47819	Glial fibrillary acidic protein	52	1.43	0.72	----
Q6IG05	Keratin_ type II cytoskeletal 75	76	1.31	0.71	----
E9PU28	Inosine-5'-monophosphate dehydrogenase 2	79	1.54	0.70	----
P09495	Tropomyosin alpha-4 chain	50	1.27	0.70	----
P25113	Phosphoglycerate mutase 1	276	1.25	0.68	----
Q3KRE8	Tubulin beta-2B chain	94	1.05	0.68	----
P14480	Fibrinogen beta chain	941	1.05	0.67	----

D3ZVU1	SprT-like domain-containing protein Spartan	72	1.13	0.67	----
P05065	Fructose-bisphosphate aldolase A	202	1.07	0.66	----
Q6P6Q2	Keratin_ type II cytoskeletal 5	76	1.16	0.66	----
Q63610	Tropomyosin alpha-3 chain	50	1.21	0.66	----
P69897	Tubulin beta-5 chain	94	1.03	0.66	----
Q4QRB4	Tubulin beta-3 chain	94	1.04	0.65	----
P08010	Glutathione S-transferase Mu 2	217	1.14	0.64	----
P55063	Heat shock 70 kDa protein 1-like	597	1.03	0.64	----
P85108	Tubulin beta-2A chain	94	1.04	0.64	----
Q6IG12	Keratin_ type II cytoskeletal 7	193	1.09	0.63	----
Q4FZU2	Keratin_ type II cytoskeletal 6A	76	1.17	0.61	----
Q62812	Myosin-9	68	1.03	0.60	----
P11980	Pyruvate kinase PKM	40	1.12	0.59	----
P62632	Elongation factor 1-alpha 2	51	1.28	0.58	----
P31232	Transgelin	9127	1.02	0.58	----
P34058	Heat shock protein HSP 90-beta	106	1.17	0.56	----
Q6P9T8	Tubulin beta-4B chain	467	1.01	0.56	----
P56574	Isocitrate dehydrogenase [NADP]_ mitochondrial	74	1.20	0.55	----
P48037	Annexin A6	120	0.99	0.52	----
P15999	ATP synthase subunit alpha_ mitochondrial	175	1.04	0.52	----
Q10758	Keratin_ type II cytoskeletal 8	193	1.04	0.52	----
P85973	Purine nucleoside phosphorylase	155	0.98	0.50	----
P14659	Heat shock-related 70 kDa protein 2	691	0.99	0.49	----
P20059	Hemopexin	409	0.99	0.46	----
P62630	Elongation factor 1-alpha 1	298	0.98	0.45	----
P70623	Fatty acid-binding protein_ adipocyte	436	0.94	0.40	----
P16409	Myosin light chain 3	787	0.95	0.40	----
P45592	Cofilin-1	297	0.96	0.38	----
P04636	Malate dehydrogenase_ mitochondrial	346	0.90	0.38	----
P04906	Glutathione S-transferase P	114	0.81	0.35	----
P07632	Superoxide dismutase [Cu-Zn]	125	0.78	0.34	----
P63018	Heat shock cognate 71 kDa protein	852	0.96	0.33	----
P62982	Ubiquitin-40S ribosomal protein S27a	274	0.82	0.31	----
P06399	Fibrinogen alpha chain	112	0.90	0.30	----
Q63429	Polyubiquitin-C	274	0.84	0.30	----
Q9JK11	Reticulon-4	39	0.27	0.30	----
Q08163	Adenylyl cyclase-associated protein 1	165	0.90	0.28	----
Q9ER34	Aconitate hydratase_ mitochondrial	83	0.86	0.27	----
P24090	Alpha-2-HS-glycoprotein	275	0.77	0.27	----
Q9WVH8	Fibulin-5	52	0.97	0.27	----
P0CG51	Polyubiquitin-B	274	0.84	0.25	----
Q07936	Annexin A2	474	0.88	0.24	----
P62986	Ubiquitin-60S ribosomal protein L40 2	274	0.83	0.24	----
P02680	Fibrinogen gamma chain	682	0.90	0.23	----
P0DMW1	Heat shock 70 kDa protein 1B	845	0.92	0.23	----
P02600	Myosin light chain 1/3_ skeletal muscle isoform	809	0.88	0.23	----
P31000	Vimentin	1733	0.96	0.21	----

P15650	Long-chain specific acyl-CoA dehydrogenase_ mitochondrial	93	0.84	0.18	----
P13437	3-ketoacyl-CoA thiolase_ mitochondrial	85	0.58	0.15	----
Q5RKI0	WD repeat-containing protein 1	192	0.89	0.15	----
Q66HD0	Endoplasmin	51	0.50	0.14	----
P12346	Serotransferrin	1002	0.90	0.13	----
P62963	Profilin-1	3064	0.90	0.11	----
P0DMW0	Heat shock 70 kDa protein 1A	849	0.86	0.10	----
Q9QXQ0	Alpha-actinin-4	431	0.88	0.08	----
Q4V8H8	EH domain-containing protein 2	225	0.61	0.06	----
P10719	ATP synthase subunit beta_ mitochondrial	87	0.84	0.05	----
P47875	Cysteine and glycine-rich protein 1	1148	0.84	0.04	↓
Q7M0E3	Destrin	1706	0.81	0.04	↓
P02454	Collagen alpha-1(I) chain	47	0.63	0.03	↓
P06866	Haptoglobin	335	0.76	0.03	↓
P50399	Rab GDP dissociation inhibitor beta	52	0.23	0.01	↓
P68136	Actin_ alpha skeletal muscle	5242	0.84	< 0.01	↓
P62738	Actin_ aortic smooth muscle	5534	0.87	< 0.01	↓
P60711	Actin_ cytoplasmic 1	4354	0.77	< 0.01	↓
P63259	Actin_ cytoplasmic 2	4358	0.77	< 0.01	↓
Q9Z1P2	Alpha-actinin-1	719	0.78	< 0.01	↓
P36201	Cysteine-rich protein 2	176	0.55	< 0.01	↓
P04797	Glyceraldehyde-3-phosphate dehydrogenase	1896	0.65	< 0.01	↓
P42930	Heat shock protein beta-1	848	0.76	< 0.01	↓
P01946	**Hemoglobin subunit alpha-1/2**	**14931**	**0.43**	**< 0.01**	↓
P02091	**Hemoglobin subunit beta-1**	**10419**	**0.32**	**< 0.01**	↓
P11517	**Hemoglobin subunit beta-2**	**3388**	**0.32**	**< 0.01**	↓
P20760	Ig gamma-2A chain C region	643	0.68	< 0.01	↓
P51886	**Lumican**	**1267**	**0.45**	**< 0.01**	↓
Q64119	Myosin light polypeptide 6	13395	0.79	< 0.01	↓
Q64122	Myosin regulatory light polypeptide 9	4053	0.74	< 0.01	↓
P10111	Peptidyl-prolyl cis-trans isomerase A	1750	0.72	< 0.01	↓
P02770	Serum albumin	7160	0.74	< 0.01	↓
P26772	10 kDa heat shock protein_ mitochondrial	124			SHR_T
B0BN56	28S ribosomal protein S31_ mitochondrial	262			SHR_T
Q7TP48	Adipocyte plasma membrane-associated protein	74			SHR_T
P17475	Alpha-1-antiproteinase	87			SHR_T
Q78E60	Aryl hydrocarbon receptor nuclear translocator 2	81			SHR_T
Q701R3	Beta-galactoside alpha-2_6-sialyltransferase 2	84			SHR_T
Q08290	Calponin-1	62			SHR_T
P11442	Clathrin heavy chain 1	27			SHR_T
P56745	Claudin-1	133			SHR_T
Q62952	Dihydropyrimidinase-related protein 3	154			SHR_T
Q9Z1Z3	Epsin-2	43			SHR_T
Q9ERW3	Fibroblast growth factor 13	84			SHR_T

P50609	Fibromodulin	241	SHR$_T$
Q9WUH4	Four and a half LIM domains protein 1	573	SHR$_T$
P10860	Glutamate dehydrogenase 1_ mitochondrial	113	SHR$_T$
P97879	Glutamate receptor-interacting protein 1	85	SHR$_T$
Q0VGK3	Glycerate kinase	118	SHR$_T$
Q5I0D1	Glyoxalase domain-containing protein 4	57	SHR$_T$
Q63406	Guanine nucleotide exchange factor DBS	29	SHR$_T$
P01835	Ig kappa chain C region_ B allele	209	SHR$_T$
D4AD37	Inositol monophosphatase 3	95	SHR$_T$
Q566E5	KDEL motif-containing protein 2	131	SHR$_T$
O35806	Latent-transforming growth factor beta-binding protein 2	66	SHR$_T$
Q5XI07	Lipoma-preferred partner homolog	119	SHR$_T$
P42123	L-lactate dehydrogenase B chain	137	SHR$_T$
Q5BK54	Lymphocyte activation gene 3 protein	78	SHR$_T$
P08494	Matrix Gla protein	185	SHR$_T$
D3ZSK5	N-lysine methyltransferase SETD6	313	SHR$_T$
Q05982	Nucleoside diphosphate kinase A	283	SHR$_T$
P19804	Nucleoside diphosphate kinase B	256	SHR$_T$
Q66HS7	PDZ and LIM domain protein 3	56	SHR$_T$
O08561	Phosphatidylinositol 4-kinase beta	26	SHR$_T$
O88422	Polypeptide N-acetylgalactosaminyltransferase 5	61	SHR$_T$
Q9ERS0	Potassium channel subfamily K member 13	71	SHR$_T$
Q9QZQ5	Protein NOV homolog	253	SHR$_T$
P83900	Rap guanine nucleotide exchange factor 5	132	SHR$_T$
I6L9G5	Reticulocalbin-3	69	SHR$_T$
D3ZA76	Serine protease HTRA3	197	SHR$_T$
P57760	Serine/threonine-protein kinase 16	155	SHR$_T$
O70142	SHC-transforming protein 2	86	SHR$_T$
Q9QWN8	Spectrin beta chain_ non-erythrocytic 2	36	SHR$_T$
P17246	Transforming growth factor beta-1 proprotein	64	SHR$_T$
P46462	Transitional endoplasmic reticulum ATPase	67	SHR$_T$
O08700	Vacuolar protein sorting-associated protein 45	128	SHR$_T$
P35213	14-3-3 protein beta/alpha	164	SHR$_C$
P62260	14-3-3 protein epsilon	148	SHR$_C$
P68511	14-3-3 protein eta	63	SHR$_C$
P61983	14-3-3 protein gamma	53	SHR$_C$
P68255	14-3-3 protein theta	164	SHR$_C$
P11884	Aldehyde dehydrogenase_ mitochondrial	90	SHR$_C$
P80299	Bifunctional epoxide hydrolase 2	47	SHR$_C$
P85125	Caveolae-associated protein 1	150	SHR$_C$

Q4V8E4	Cilia- and flagella-associated protein 36	83	SHR_C
Q68FY0	Cytochrome b-c1 complex subunit 1_ mitochondrial	160	SHR_C
Q01129	Decorin	150	SHR_C
O55096	Dipeptidyl peptidase 3	45	SHR_C
Q91XQ4	DNA-directed RNA polymerase II subunit GRINL1A	69	SHR_C
Q62862	Dual specificity mitogen-activated protein kinase kinase 5	67	SHR_C
Q5EZ72	Ectonucleotide pyrophosphatase/phosphodiesterase family member 7	42	SHR_C
Q68FU3	Electron transfer flavoprotein subunit beta	536	SHR_C
Q66H04	F-box only protein 43	68	SHR_C
P09117	Fructose-bisphosphate aldolase C	79	SHR_C
P13255	Glycine N-methyltransferase	44	SHR_C
Q5XHZ0	Heat shock protein 75 kDa_ mitochondrial	47	SHR_C
Q00715	Histone H2B type 1	122	SHR_C
A6YP92	Homeobox protein ARX	33	SHR_C
P20759	Ig gamma-1 chain C region	31	SHR_C
Q6IE24	Inactive ubiquitin carboxyl-terminal hydrolase 54	36	SHR_C
Q99J82	Integrin-linked protein kinase	75	SHR_C
P18588	Interferon-induced GTP-binding protein Mx1	61	SHR_C
P15800	Laminin subunit beta-2	47	SHR_C
Q5U2U7	mRNA cap guanine-N7 methyltransferase	99	SHR_C
A7E3N2	Neutrophil cytosol factor 2	52	SHR_C
P16617	Phosphoglycerate kinase 1	73	SHR_C
P11506	Plasma membrane calcium-transporting ATPase 2	71	SHR_C
O88767	Protein/nucleic acid deglycase DJ-1	229	SHR_C
P50398	Rab GDP dissociation inhibitor alpha	112	SHR_C
P42346	Serine/threonine-protein kinase mTOR	52	SHR_C
P01048	T-kininogen 1	55	SHR_C
P50137	Transketolase	50	SHR_C

[a] identification of proteins in the Uniprot database (http://www.uniprot.org/).
[b] Significant difference in expression proteins up regulation, down regulation and intermediates.
[c] p value ($p<0.05$) .
[d] Up regulated (↑), Intermediate (----, present in both comparison groups), down regulated (↓) and unique each comparison group (SHR_T and SHR_C).

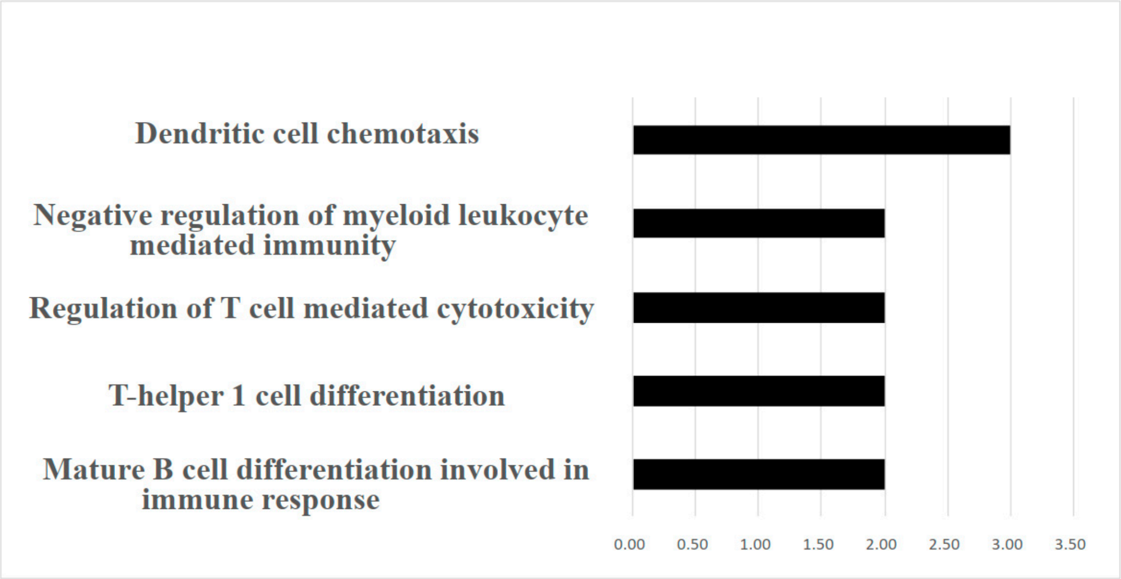

Figure S1: Protein analysis in ClueGO plugins and the number of genes involved within the sistem immune category between SHR_P vs. SHR_C groups.

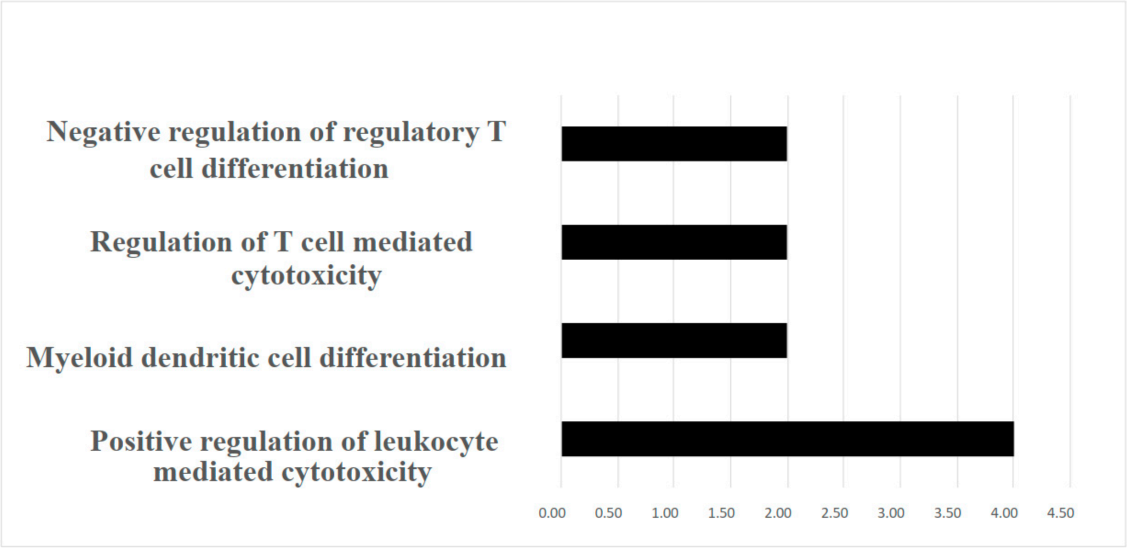

Figure S2 : Protein analysis in ClueGO plug-ins and the number of genes involved within the sistem immune category between SHR_T vs. SHR_C groups
